# Regulation of MRE11A by UBQLN4 leads to cisplatin resistance in patients with esophageal squamous cell carcinoma

**DOI:** 10.1002/1878-0261.12929

**Published:** 2021-03-08

**Authors:** Tomohiro Murakami, Yoshiaki Shoji, Tomohiko Nishi, Shu‐Ching Chang, Ron D. Jachimowicz, Sojun Hoshimoto, Shigeshi Ono, Yosef Shiloh, Hiroya Takeuchi, Yuko Kitagawa, Dave S. B. Hoon, Matias A. Bustos

**Affiliations:** ^1^ Department of Translational Molecular Medicine Division of Molecular Oncology Saint John’s Cancer Institute at Providence Saint John’s Health Center Santa Monica CA USA; ^2^ Department of Surgery Hamamatsu University School of Medicine Japan; ^3^ Department of Surgery Keio University School of Medicine Shinjuku‐ku Japan; ^4^ Medical Data Research Center Providence Health and Services at Providence Saint Joseph’s Health Portland OR USA; ^5^ Clinic I of Internal Medicine University Hospital Cologne Germany; ^6^ Max Planck Institute for Biology of Ageing Cologne Germany; ^7^ Center for Molecular Medicine Cologne University of Cologne Germany; ^8^ Cologne Excellence Cluster on Cellular Stress Response in Ageing‐Associated Diseases University of Cologne Germany; ^9^ David and Inez Myers Laboratory for Cancer Genetics Sackler School of Medicine Tel Aviv University Israel

**Keywords:** chemoresistance, esophageal cancer, MRE11, neoadjuvant chemotherapy, ubiquilin‐4

## Abstract

Resistance to standard cisplatin‐based chemotherapies leads to worse survival outcomes for patients with esophageal squamous cell carcinoma (ESCC). Therefore, there is an urgent need to understand the aberrant mechanisms driving resistance in ESCC tumors. We hypothesized that ubiquilin‐4 (UBQLN4), a protein that targets ubiquitinated proteins to the proteasome, regulates the expression of Meiotic Recombination 11 Homolog A (MRE11A), a critical component of the MRN complex and DNA damage repair pathways. Initially, immunohistochemistry analysis was conducted in specimens from patients with ESCC (*n* = 120). In endoscopic core ESCC biopsies taken from 61 patients who underwent neoadjuvant chemotherapy (NAC) (5‐fluorouracil and cisplatin), low MRE11A and high UBQLN4 protein levels were associated with reduced pathological response to NAC (*P* < 0.001 and *P* < 0.001, respectively). Multivariable analysis of surgically resected ESCC tissues from 59 patients revealed low MRE11A and high UBLQN4 expression as independent factors that can predict shorter overall survival [*P = *0.01, hazard ratio (HR) = 5.11, 95% confidence interval (CI), 1.45–18.03; *P = *0.02, HR = 3.74, 95% CI, 1.19–11.76, respectively]. Suppression of MRE11A expression was associated with cisplatin resistance in ESCC cell lines. Additionally, MRE11A was found to be ubiquitinated after cisplatin treatment. We observed an amplification of *UBQLN4* gene copy numbers and an increase in UBQLN4 protein levels in ESCC tissues. Binding of UBQLN4 to ubiquitinated‐MRE11A increased MRE11A degradation, thereby regulating MRE11A protein levels following DNA damage and promoting cisplatin resistance. In summary, MRE11A and UBQLN4 protein levels can serve as predictors for NAC response and as prognostic markers in ESCC patients.

## Graphical abstract

We unraveled a novel mechanism driving resistance to cisplatin‐based chemotherapies in esophageal squamous cell carcinoma (ESCC) and demonstrated their clinical utility. Briefly, we showed that cisplatin treatment promotes Meiotic Recombination 11 Homolog A (MRE11A) ubiquitination. Binding of ubiquilin‐4 (UBQLN4) to ubiquitinated‐MRE11A increased MRE11A degradation, thereby promoting cisplatin resistance. Both MRE11A and UBQLN4 can predict neoadjuvant chemotherapy response and serve as prognostic markers in ESCC patients.

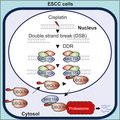

**Abbreviations**
Ab: antibody
AJCC: American Joint Committee on Cancer
Co‐IP: Co‐Immunoprecipitation
DSB: double‐strand break
EAC: esophageal adenocarcinoma
ESCA: esophageal carcinoma
ESCC: esophageal squamous cell carcinoma
EV: empty vector
FFPE: formalin‐fixed paraffin‐embedded
IF: immunofluorescence
IHC: immunohistochemistry
mAb: monoclonal antibody
MRE11A: Meiotic Recombination 11 Homolog A
MRN: MRE11‐RAD50‐NBS1
NAC: neoadjuvant chemotherapy
NS: not significant
OS: overall survival
OV: overexpression
pAb: polyclonal antibody
SD: standard deviation
TCGA: The Cancer Genome Atlas
UBA: ubiquitin‐associated domain
Ubl: ubiquitin‐like domain
UBQLN4: ubiquilin‐4

## 1 Introduction

Esophageal cancer represents the 7th cause of cancer death globally [1]. The two major histological types that account for over 90% are esophageal squamous cell carcinoma (ESCC) and esophageal adenocarcinoma (EAC) [2]. ESCC is the most common histology worldwide (~ 90%), with a particularly high incidence across Asia and southeastern Africa while being equally frequent to EAC (~ 50%) in northern America and Europe [3, 4, 5]. In locally advanced ESCCs, neoadjuvant chemotherapy (NAC) improves overall survival (OS) when compared to surgery alone [6] or postoperative adjuvant treatment [7], and platinum‐based drugs stand as key agents in treatment regimens combined with fluorouracil and taxanes [7, 8, 9]. Despite the clinical advances in ESCC chemotherapy, the response rates remain as low as 40% [10]. Up to 80% of ESCC patients experience recurrence leading to early mortality [11] and the 5‐year OS remains ~ 20% [12]. Importantly, patients who have a better pathological response to NAC have a better OS and disease‐free survival [13, 14, 15]. However, only a few biomarkers have been used to predict response to NAC, with their clinical relevance still being underestimated [16]. Therefore, in order to improve patient outcomes, it is critical to understand the molecular basis of the tumor progression in ESCC tumors and identify aberrant DNA–damage response (DDR) mechanisms that drive resistance to current drugs used in NAC.

The MRE11‐RAD50‐NBS1 (MRN) complex has an important role in DDR and chemotherapy treatment. MRN complex sensors and orchestrates DDR in double‐strand breaks (DSB), stalled replication forks, dysfunctional telomeres, and immune responses [17, 18]. Alterations of MRN complex have been associated with chemotherapy and radiotherapy resistance [18, 19, 20, 21]. There is an assumption that the upregulation of the MRN complex is associated with the resistance to DNA damaging therapy due to the higher efficiency of DNA damage repair [17, 19, 20, 21, 22]. However, there is still controversy whether this assumption can be generalized to all tumor types as some differences may arise from different tumor origin. In regards, it was previously shown that certain cancer types are less susceptible to DNA damaging therapies when they have lower expression of the MRN complex components [23, 24, 25]. The mechanisms controlling MRN complex, in particular Meiotic Recombination 11 Homolog A (MRE11A) as related to ESCC chemoresistance, are not fully understood.

Ubiquilin family proteins function as adaptors between ubiquitinated proteins and the proteasome [26, 27, 28, 29]. Five ubiquilin genes (UBQLN1‐4 and UBQLNL) have been identified in the human genome [28]. Ubiquilins contain two conserved domains: Ubiquitin‐like domain (Ubl) and Ubiquitin‐associated domain (UBA). The Ubl domain has homology to the ubiquitin domain and interacts with the proteasome regulatory component s5a, while the UBA domain binds to mono‐ and poly‐ubiquitin [30]. UBQLN4 was recently shown to have a role to shuttle nuclear proteins to the cytosol for degradation through the nuclear pore [31]. Studies have shown that UBQLN4 targets misassembled ER‐localized proteins to the proteasome, thus reducing proteotoxic cell stress and harboring a protective role [26]. However, there is no evidence showing whether UBQLN4 has a role in reducing genotoxic stress in ESCC tumor cells during chemotherapy treatment and how that affects the tumor sensitivity to chemotherapy.

Although hypomorphic mutations on MRE11A have been identified in different solid tumors [17], mutations in *MRE11A* gene are rare in ESCC tumors. UBQLN4 has a role in controlling nuclear protein levels by targeting ubiquitinated proteins to the proteasome [29, 31]. In ESCC, we proposed the hypothesis that during DNA damage MRE11A is degraded by the ubiquitin–proteasome system facilitated by UBQLN4. The aims of this study were as follows: (a) to unravel the regulatory mechanism affecting MRE11A; (b) determine the role of MRE11A in controlling the response to cisplatin‐based therapy in ESCC patients; and (c) analyze the associations between MRE11A expression and OS in patients with ESCC.

## 2 Materials and methods

### 2.1 ESCC patient’s tissues

Formalin‐fixed paraffin‐embedded (FFPE) from 61 endoscopic biopsy specimens prior to NAC were obtained from clinical stage II/III [American Joint Committee on Cancer (AJCC) 8th ed.] ESCC patients who underwent NAC followed by surgery at Keio University Hospital, Department of Surgery, Tokyo, Japan, between 2011 and 2016. These patients did not undergo adjuvant treatment. Specimens met the following criteria: (a) histologically proven ESCC, (b) no previous history of chemotherapy or chemoradiotherapy for any malignancies, and (c) no microscopic residual tumor (R0). For NAC, cisplatin plus 5‐fluorouracil was repeated twice every 3 weeks (day 1: cisplatin by 80 mg·m^−2^; days 1–5: 5‐fluorouracil by 800 mg·m^−2^) [7]. Tumor regression grade was evaluated by pathologists at Keio University Hospital. Responders are defined as marked changes in two‐thirds or more in the tumor area. Nonresponders were defined as marked changes in less than two‐third. Written informed consent was obtained from all subjects. Immunohistochemistry (IHC) for MRE11A and UBQLN4 was performed for this cohort and IHC was quantified to obtain *H*‐score values. Associations between their *H*‐scores and treatment response to NAC were then analyzed.

Formalin‐fixed paraffin‐embedded tissues from 59 surgically resected ESCC primary tumors were obtained from patients who underwent up‐front surgery at Keio University Hospital from 1997 to 2002 [32]. IHC for MRE11A and UBQLN4 was also performed and IHC was quantified to obtain the *H*‐score values. Univariate and multivariate analysis was performed to determine associations between *H*‐scores and clinical variables.

All of the experiments followed World Medical Association Declaration of Helsinki and the NIH Belmont Report principles. Tissue specimens were coded according to HIPAA recommendation. This study was approved by Keio University School of Medicine Ethics Committee with a registration number of 2016‐0233 and 2016‐241. All human samples and clinical information for this study were obtained according to the protocol guidelines approved by the Joint Institutional Review Board of Providence Saint John's Health Center/ Saint John's Cancer Institute and the Western Institutional Review Board (USA).

### 2.2 Cell lines

Established human ESCC cell lines TE‐4 (CVCL_3337), TE‐8 (CVCL_1766), and TE‐10 (CVCL_1760) were obtained from Keio University. Cell lines were cultured in Dulbecco's modified Eagle's medium or RPMI supplemented with 10% FBS at 37 °C with 5% CO_2_. All human cell lines have been authenticated using short tandem repeat profiling within the last 3 years. All experiments were performed with mycoplasma‐free cell lines.

### 2.3 Plasmids and ESCC cisplatin‐resistant cell lines

MRE11A EX‐q‐0491‐M35 and the empty control vector EX‐EGFP‐M35 were from Genecopoeia, Rockville, MD, USA; ubiquitin B pCMV6 vector and the empty vector (EV) were from Origene, Rockville, MD, USA; UBQLN4 was cloned in pLNCX2 plasmid as described previously [29]. For stable clones, UBQLN4 or EVs were packaged into lentivirus particles using HEK‐293T cell lines. Purified lentiviral particles were transduced into TE‐8 and TE‐10 cell lines. Stable clones were selected with G418 100 μg·mL^−1^.

For establishment of cisplatin‐resistant ESCC cell lines, TE‐8 and TE‐10 cell lines were cultured in 2 μm of cisplatin (#S1166; Selleck Chemicals, Houston, TX, USA) supplemented medium for 3 days. Afterward, the medium was replaced and the cell lines were cultured until the resistant clones proliferated; the treatment was repeated with 3 μm and then 5 μm of cisplatin supplemented medium. Then, the cell lines were continuously cultured in 2.5 μm of cisplatin for 4 weeks. Cell lines were recovered in cisplatin‐free medium and used for further assays. For knockdown experiments, TE‐4 cell lines were transfected with 20 nm pool siRNA UBQLN4 or nontargeting Pool (L‐021178‐01‐0005 and D‐001810‐10‐05, respectively; Dharmacon, Lafayette, CO, USA) using jetPRIME Polyplus transfection (#114‐15; Polypus Transfection, Illkirch, France). TE‐8 and TE‐10 cell lines were transfected with 50 nm pool siRNA MRE11A or nontargeting Pool (L‐009271‐00‐0005 and D‐001810‐10‐05, respectively; Dharmacon) using jetPRIME Polyplus transfection.

### 2.4 Immunohistochemistry (IHC)

Endoscopic biopsy specimens and surgically resected ESCC tumors were stained with UBQLN4 monoclonal antibody (mAb; #sc‐136145; Santa Cruz Biotechnology, Santa Cruz, CA, USA) and/or MRE11A polyclonal antibody (pAb; #4895S; Cell Signaling Technology, Danvers, MA, USA) as previously described [33]. Images were taken by the BX43 upright microscope (Olympus, Tokyo, Japan) with a magnification of 20× using the mantra snap Software 1.03 (Perkin Elmer, Waltham, MA, USA). *H*‐scores were calculated using the inform 2.4 software (Perkin Elmer) and following manual instructions. Briefly, the tumor area was segmented from the stromal area and nuclei/cytoplasm compartments were distinguished by detecting the intensity of hematoxylin and/or 3,3'‐diaminobenzidine (DAB) staining. The optical signal threshold to classify the score into 4‐bins was set to 0.05, 0.12, and 0.2. Five slides per case were analyzed and the average *H*‐score was then used as the final value. The cutoff values for UBQLN4 and MRE11A were determined by considering *H*‐score values for the mean value observed in normal adjacent epithelia esophagus tissues plus 10 SD.

### 2.5 Co‐Immunoprecipitation assays

Cell lines were transfected with MRE11A and UBB plasmids as indicated in each experiment. After 24 h, cell lines were treated with 5 μm MG‐132 (#S2619; Selleck chemicals) and/or 5 μm cisplatin for 12 h. After incubation, cell lines were washed and lysed in Co‐Immunoprecitation (Co‐IP) buffer (150 mm NaCl, 100 mm Tris/HCl pH 8, 1% NP‐40, protease, and phosphatase inhibitors) by gently pipetting. The whole‐cell (WC) lysates were quantified by bicinchoninic acid assay, and 250 μg of WC lysate (final concentration 1.25 μg·μL^−1^) was incubated with UBQLN4 mAb (#sc‐136145; Santa Cruz Biotechnology) and mouse (G3A1) mAb control (#5415; Cell Signaling Technology); or UBQLN4‐Ps318 pAb (#A300‐L20645, Bethyl) and mouse (G3A1) mAb control (#5415; Cell Signaling Technology); or MRE11A pAb (#4895S; Cell Signaling Technology) and normal rabbit pAb control (#2729S; Cell Signaling Technology); or DDDDK tag pAb(#ab1162; Abcam, Cambridge, MA, USA) and normal rabbit pAb control (#2729S; Cell Signaling Technology) in Co‐IP buffer. Immunocomplex was further incubated with Dynabeads™ Protein A (#10002D; ThermoFisher Scientific, Waltham, MA, USA) for 1 h at 4 °C. Only for the experiment showed in Fig. S3A, we incubated the beads from all the conditions with buffer or 50 nm of recombinant human USP2 catalytic domain protein (Bio‐Techne Corporation, Minneapolis, MN, USA) for 30 min at 37 °C, as indicated. Beads were washed three times with Co‐IP buffer, recovered, and boiled in Fluorescent Master Mix for 5 min at 95 °C in a dry bath. After centrifugation, the supernatants (SN) were recovered and protein concentration was adjusted to 1 μg·μL^−1^ with 1× MM reagent. Samples were analyzed by automated western blot system [33, 34].

### 2.6 On‐chip cell sorting to purify UBQLN4‐GFP cell lines

The microfluidic chip‐based cell sorter, On‐chip Sort (On‐chip Biotechnologies, Tokyo, Japan), equipped with blue (488 nm), violet (405 nm), and red (637 nm) lasers, was used according to the manufacturer's protocol. TE‐8 and TE‐10 UBQLN4‐OV cell lines were harvested, centrifuged, and diluted to 1 × 10^7^/mL. The sorting microfluidic chip was precoated with On‐chip T‐buffer before loading the sample. After priming the chip, excessive T‐buffer was removed from all reservoirs, and replaced with fresh culture medium. Then, the prepared samples were loaded. Gates were set and the samples were sorted according to the cell size and GFP positivity of interest. The sorted samples were retrieved, and the sorting process was repeated three times to increase the purity of the sorted cell lines. After sorting, cell lines were immediately cultured until 80% confluence and then frozen.

### 2.7 Immunofluorescence (IF)

Cell lines (1–1.5 × 10^4^) were seeded in 8‐well Falcon™ chambered culture slides (Fisher Scientific) and incubated for 48 h, followed by treatment with 5 μm cisplatin for 12 h. After that, cell lines were fixed and the protocol was followed as described in [34] except that primary Ab dilutions in 5% BSA are as shown in Table S1. The photographs were acquired using a Nikon Eclipse Ti microscope and NIS elements software. Quantification of fluorescent signal was performed on imagej software (ImageJ; NIH, Bethesda, MD, USA).

### 2.8 Cell viability and colony formation assays

ESCC cell lines (1–2 × 10^3^) were seeded in a 96‐well plate. The number of viable cells was assessed using a Cell Titer‐Glo Luminescent (Promega, Madison, WI, USA) according to the manufacturers' instructions. For colony formation, ESCC cell lines (2 × 10^3^) were seeded in a 6‐well plate and after 12 days of incubation processed as previously described [34].

### 2.9 Cycloheximide chase assays

TE‐10 cell lines (80% confluency) were incubated with 200 μg·mL^−1^ of cycloheximide (#C7698; Millipore Sigma, Darmstadt, Germany) with or without 5 μm MG‐132 at different time points (0, 20, 40, and 60 min). After incubation times, cell lines were harvested and processed for protein extraction. Western blot for MRE11A and β‐actin was performed and their expression was quantified over time and adjusted to β‐actin levels [33, 34, 35].

### 2.10 Nuclear extraction

Nuclear and cytoplasmic fractions were isolated from the TE‐10 and TE‐8 cell line with the Nuclear Extract Kit (Active Motif, Carlsbad, CA, USA). Cells (8.8 × 10^6^ cells/dish) were cultured in 100‐mm dishes and harvested with 3 mL cold PBS/phosphatase inhibitor buffer. Cells were centrifuged and the WC pellet was gently suspended in 500 µL 1× hypotonic buffer and incubated for 15 min on ice. Then, 25 µL of detergent was added to induce cell lysis. After cell lysis, the cytoplasmic fraction (supernatant) was separated from the nuclear fraction (pellet) by centrifugation (for 30 s at 14 000 ***g***). Then, the nuclear fraction (pellet) was resuspended in 50 µL of the complete lysis buffer and incubated with 2.5 µL detergent for 30 min on ice. The nuclear lysates were centrifuged for 10 min at 14 000 ***g***. The nuclear fraction (supernatant) was then collected. Both nuclear and cytoplasmic fractions were analyzed by automated western blot.

### 2.11 Western blot

Automated western blot was performed according to the manufacturer's protocol (Protein Simple, San Jose, CA, USA; https://www.proteinsimple.com/wes.html [36, 37]). Briefly, the proteins were extracted with lysis buffer (150 mm NaCl, 100 mm Tris/HCl pH 8, 1% NP‐40, and protease inhibitors) and the protein concentration was adjusted to 0.5–1 μg·μL^−1^. The results were analyzed using the Compass Software (Protein Simple) and imagej software (ImageJ; NIH). The primary Ab dilutions are shown in Table S1. All uncropped western blot images are shown in Fig. S7,S8.

### 2.12 Biostatistical and bioinformatics analysis

Normal distribution of data was tested using the Kolmogorov–Smirnov test. The parametric Student's *t*‐test was used for normally distributed data, and the nonparametric Wilcoxon rank‐sum test was used for non‐normally distributed data. One‐way ANOVA test and Bonferroni *post hoc* test was applied in the quantification of IF assays. Two‐way ANOVA test and the Bonferroni *post hoc* test were applied for cell proliferation and drug sensitivity analysis. Correlation analyses were performed using Pearson's or Spearman's correlation coefficient (*r*) according to the data distribution. OS was compared using the Kaplan–Meier method and log‐rank test. Multivariable analysis was performed by the Cox proportional regression model to analyze the independent predictor for OS. Significant factors considering the covariate factors and age were included for the multivariable analysis. A two‐sided *P* < 0.05 was considered statistically significant: *P < 0.05; **P < 0.01; ***P < 0.001 and NS, not significant. All analyses were performed with graphpad prism 5 (GraphPad software Inc., La Jolla, CA, USA) or r version 3.5.0 (R Core Team, 2018 [38]), and the figures were made using CorelDraw graphics suite 8× (Corel Corporation, Ottawa, ON, Canada).

## 3 Results

### 3.1 MRE11A is upregulated in ESCCs and loss of MRE11A leads to cisplatin resistance

**Fig. 1:**
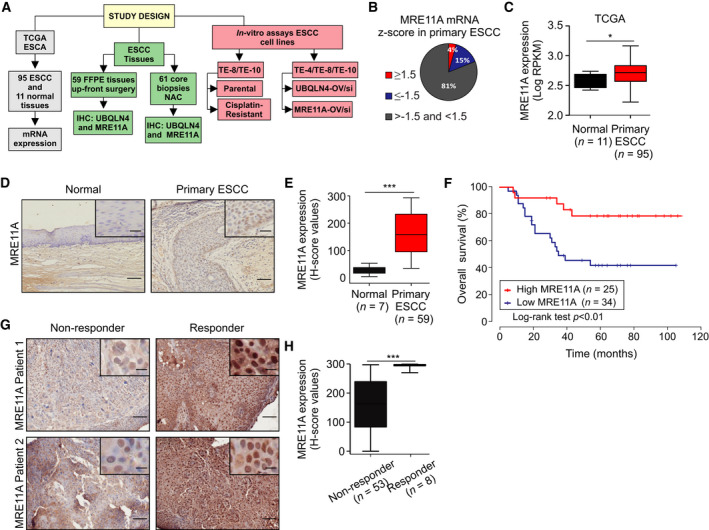
Loss of MRE11A leads to chemotherapy resistance. (A) Scheme of the study design. (B) Pie chart showing the proportion of ESCC cases with high or low *MRE11A* in the TCGA ESCA database. ESCC patients were divided according to the *z*‐score values into 1) ≥ 1.5, 2) ≤ −1.5, or 3) < 1.5 and > −1.5 for *MRE11A* mRNA expression levels. (C) *MRE11A* mRNA expression levels in normal adjacent esophageal epithelia (*n* = 11) and primary ESCC tumors (*n* = 95) in the TCGA ESCA database (*P = 0.05). (D) Representative images of normal adjacent esophageal epithelia and primary ESCC tumors showing MRE11A protein levels stained for MRE11A using IHC. Scale bars = 50 µm. Right top insets on each picture show a magnification of MRE11A staining. Scale bars = 10 µm. (E) Comparison of *H*‐scores for MRE11A protein levels for normal adjacent esophageal epithelia (*n* = 7) and primary ESCC tumor tissues (*n* = 59) (***P < 0.001). (F) Kaplan–Meier curves comparing OS in ESCC patients with low (*n* = 34) versus high (*n* = 25) MRE11A protein levels (*P < *0.01). (G) Representative images of core biopsy tissues from nonresponders (Patients 1 and 2) and responders (Patients 1 and 2) ESCC patients to NAC that were stained for MRE11A using IHC. Scale bars = 50 µm. Right top insets on each picture show a magnification of MRE11A staining. Scale bars = 10 µm. (H) Comparison of *H*‐scores for MRE11A protein levels in core biopsy tissues from nonresponders (*n* = 53) or responders (*n* = 8) ESCC patients to NAC (***P < 0.001). Error bars represent the mean ± SD. Statistical differences were tested using Mann–Whitney test (C), an unpaired two‐tailed *t*‐test with Welch’s correction (E and H) and log‐rank test (F).

*MRE11A* expression was analyzed in the The Cancer Genome Atlas (TCGA) esophageal carcinoma (ESCA) dataset [39]. This database contains both EAC and ESCC (*n* = 186) of which all 95 ESCCs were analyzed (Fig. 1A‐C). *MRE11A* was highly expressed (mRNA *z*‐score ≥ 1.5) in about 4% (4 of 95) and reduced in 15% (*z*‐score ≤ −1.5) of ESCC patients (Fig. 1B). In addition, *MRE11A* expression was significantly higher in primary ESCC tumor tissues compared to normal adjacent esophageal epithelia tissues (Fig. 1C).

To further elucidate the clinical significance of MRE11A, IHC was performed and *H*‐score values were obtained from surgically resected ESCC tissues (FFPE) clinically annotated (Table 1). Normal adjacent esophageal epithelia showed lower *H*‐score values for MRE11A (*n* = 7, *H*‐score = 29.9 ± 14.5) compared to primary ESCC tumors (*n* = 59, *H*‐score = 163.4 ± 75.3, *P* < 0.0001, Fig. 1D,E). A cutoff *H*‐score value > 180 was considered as high for MRE11A IHC staining in primary ESCC (Fig. S1A). Patients with low MRE11A expression (median 5 years OS = 42 months, *n* = 34) had a worse prognosis compared to those with high MRE11A expression (median 5 years OS = 79 months, *n* = 25, *P* < 0.01, Fig. 1F). Strikingly, multivariable analysis revealed that MRE11A was an independent factor to predict OS [*P = *0.01, hazard ratio (HR) = 5.11, 95% confidence interval (CI) 1.45–18.03, Table 2].

**Table 1:** Clinicopathological features for primary ESCC patients (*n* = 60). NA, not applicable.

Age, median (Q1, Q3)	60.0 (56, 64.0)
Age, *n* (%)	
< 60	23 (39.0)
≥ 60	27 (45.8)
NA	9 (15.2)
Gender, *n* (%)	
Male	54 (91.5)
Female	5 (8.5)
Differentiation, *n* (%)	
Well	14 (23.7)
Moderate / poor	45 (76.3)
T stage, *n* (%)	
1,2	33 (55.9)
3	26 (44.1)
N stage, *n* (%)	
0	20 (33.9)
1	39 (66.1)
M stage, *n* (%)	
0	59 (100)
AJCC8th pathological stage, *n* (%)	
I,II	31 (52.5)
III,IV	28 (47.5)
Lymphatic invasion, *n* (%)	
Negative	12 (20.3)
Positive	47 (79.7)
Vascular invasion, *n* (%)	
Negative	33 (55.9)
Positive	26 (44.1)
Mortality, *n* (%)	
Alive	36 (61)
Death	23 (39)
UBQLN4 *H*‐score, median (Q1, Q3)	81 (8.1, 134.6)
UBQLN4 *H*‐score (cutoff = 110), *n* (%)	
Low	39 (66.1)
High	20 (33.9)
MRE11A *H*‐score, median (Q1, Q3)	158.8 (97.1, 232.3)
MRE11A *H*‐score (cutoff = 180), *n* (%)	
Low	34 (57.6)
High	25 (42.4)

**Table 2:** Univariable and multivariable analysis for clinicopathological features in relation to OS in ESCC patients (*n* = 60).

	Univariable analysis				Multivariable analysis			
	HR	Lower 95% CI	Upper 95% CI	*P*‐value	HR	Lower 95% CI	Upper 95% CI	*P*‐value
Age, continuous	1.01	0.93	1.07	0.98	1.02	0.96	1.09	0.47
Age								
< 60	1							
≥ 60	1.49	0.58	3.86	0.41				
Gender								
Male	1							
Female	0.55	0.07	4.05	0.55				
Differentiation								
Well	1							
Moderate/poor	1.65	0.56	4.87	0.36				
T stage								
1, 2	1							
3	3.36	1.44	7.83	0.01				
N stage								
0	1							
1	3.52	1.19	10.38	0.02				
AJCC8th pathological stage								
I,II	1				1			
III,IV	4.11	1.68	10.08	0.002	1.5	0.43	5.18	0.52
Lymphatic invasion								
Negative	1				1			
Positive	3.77	0.88	16.01	0.07	2.48	0.27	22.66	0.42
Vascular invasion								
Negative	1				1			
Positive	2.65	1.15	6.11	0.02	2.33	0.7	7.72	0.17
MRE11A *H*‐score, continuous	0.99	0.99	1	0.02				
MRE11A *H*‐score (cutoff = 180)								
High	1				1			
Low	3.66	1.35	9.91	0.01	5.11	1.45	18.03	0.01
UBQLN4 *H*‐score, continuous	1.01	1	1.01	0.002				
UBQLN4 *H*‐score (cutoff = 110)								
Low	1				1			
High	2.74	1.2	6.26	0.02	3.74	1.19	11.76	0.02

Then, MRE11A expression was assessed by IHC on 61 endoscopic biopsy FFPE tissues procured from patients with AJCC Stage II/III ESCC, who underwent cisplatin‐based NAC followed by surgery. The biopsies were collected before NAC treatment. Then, the patients were stratified based on pathological response to NAC into responder and nonresponders. *H*‐score values demonstrated that MRE11A expression was significantly increased in responder (*n* = 8) compared to nonresponder ESCC patients who underwent NAC (*n* = 53; *H*‐score = 293.3 ± 3.4 versus 162.8 ± 11.8, *P* < 0.001, Fig. 1G,H).

### 3.2 MRE11A expression determines cisplatin resistance in ESCC cell lines

**Fig. 2:**
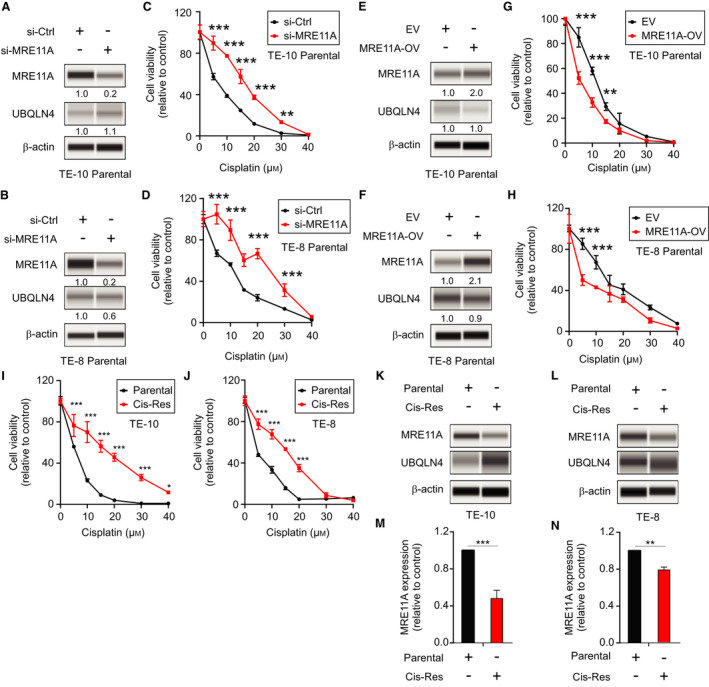
MRE11A expression determines cisplatin resistance in ESCC cell lines. (A, B) Western blot analysis for MRE11A, UBQLN4, and β‐actin (loading control) comparing si‐Ctrl and si‐MRE11A in TE‐10 (A) and TE‐8 (B) parental cell lines. (C, D) Drug sensitivity assays comparing si‐Ctrl or si‐MRE11A in TE‐10 (C) and TE‐8 (D) parental cell lines treated with different cisplatin concentrations (**P < 0.01, ***P < 0.001). (E, F) MRE11A, UBQLN4, and β‐actin (loading control) comparing EV and MRE11A‐OV in TE‐10 (E) or TE‐8 (F) parental cell lines. (G, H) Drug sensitivity assays for TE‐10 (G) and TE‐8 (H), EV or MRE11A‐OV parental cell lines treated with different cisplatin concentrations (**P < 0.01, ***P < 0.001). (I, J) Drug sensitivity assays for TE‐10 (I) and TE‐8 (J) parental or established cisplatin‐resistant (Cis‐Res) cell lines treated with different cisplatin concentrations (*P < 0.05, ***P < 0.001). (K, L) Western blot analysis for MRE11A, UBQLN4, and β‐actin (loading control) comparing parental and cisplatin‐resistant (Cis‐Res) TE‐10 (K) or TE‐8 (L) ESCC cell lines. (M, N) Quantification of MRE11A protein levels analyzed by western blot comparing parental and cisplatin‐resistant (Cis‐Res) TE‐10 (M) or TE‐8 (N) ESCC cell lines (**P < 0.01, ***P < 0.001). Error bars represent the mean ± SD from *n* = 3 replicates. Statistical differences were tested using two‐way ANOVA test and *post hoc* Bonferroni test (C, D, G, H, I, and J) and unpaired two‐tailed *t*‐test (M and N).

To determine the role of MRE11A in controlling cisplatin sensitivity, we performed MRE11A knockdown and overexpression (OV) in ESCC cell lines and analyzed them for cisplatin sensitivity. MRE11A knockdown promoted cisplatin resistance in ESCC cell lines (Fig. 2A–D). Conversely, MRE11A‐OV enhanced cisplatin sensitivity in ESCC cell lines (Fig. 2E–H).

In order to determine whether MRE11A was associated with cisplatin resistance, we established two cisplatin‐resistant ESCC cell lines. Cisplatin resistance in ESCC cell lines was confirmed by drug sensitivity assays (Fig. 2I,J). In agreement with our previous observation, western blot analysis revealed that MRE11A expression was significantly lower in the cisplatin‐resistant compared to respective parental cell lines (Fig. 2K–N). To further validate this observation, cisplatin‐resistant cell lines were knockdown for MRE11A (Fig. S2A,B) and assessed for the sensitivity to cisplatin. The results showed that cisplatin‐resistant cell lines with MRE11A knockdown were even more resistant to cisplatin compared to the respective parental cell lines (Fig. S2C,D). Conversely, cisplatin‐resistant cell lines with MRE11A‐OV (Fig. S2E,F) were tested for cisplatin sensitivity. MRE11A‐OV in cisplatin‐resistant cell lines restored the sensitivity to cisplatin (Fig. S2G,H). These results suggested that MRE11A expression controls cisplatin resistance in ESCC cell lines.

### 3.3 UBQLN4 promotes MRE11A degradation

**Fig. 3:**
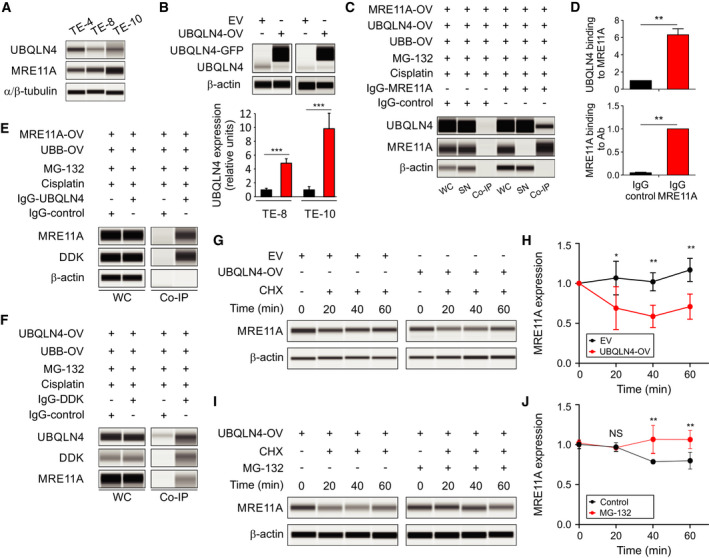
UBQLN4 binds to ubiquitinated‐MRE11A and promotes MRE11A degradation. (A) TE‐4, TE‐8, and TE‐10 cell lines were profiled for UBQLN4, MRE11A, and α/β‐tubulin (loading control). (B) TE‐8 and TE‐10 clones with EV or UBQLN4‐OV were analyzed by western blot for UBQLN4 and β‐actin (loading control) (***P < 0.001). (C) Co‐IP assay in TE‐10 UBQLN4‐OV cell lines that were treated with cisplatin (5 μm) and MG‐132 (5 μm) using anti‐MRE11A IgG Ab or control IgG Ab. UBQLN4, MRE11A, and β‐actin (loading control) protein levels were assessed in WC lysates, SN, or Co‐IP fractions (Co‐IP). (D) Quantification of UBQLN4 and MRE11A in the Co‐IP fractions (**P < 0.01). (E) Co‐IP assay in TE‐4 cell lines overexpressing MRE11A and UBB that were treated with cisplatin (5 μm) and MG‐132 (5 μm) using anti‐UBQLN4 IgG Ab or control IgG Ab. MRE11A, DDK tag, and β‐actin (loading control) protein levels were assessed in WC lysates and Co‐IP fractions (Co‐IP). (F) Co‐IP assay in TE‐10 UBQLN4‐OV and UBB‐OV cell lines that were treated with cisplatin (5 μm) and MG‐132 (5 μm) using anti‐DDK IgG Ab or control IgG Ab. UBQLN4, DDK tag, and MRE11A protein levels were assessed in WC lysates and Co‐IP fractions (Co‐IP). (G) Cycloheximide chase assay in TE‐10 EV and UBQLN4‐OV cell lines. Western blot analysis was performed for MRE11A and β‐actin (loading control). (H) Quantification for MRE11A protein levels on the cycloheximide chase assay (*P < 0.05, **P < 0.01). (I) Cycloheximide chase assay in TE‐10 UBQLN4‐OV cell lines with or without MG‐132 (5 μm). Western blot analysis was performed for MRE11A and β‐actin (loading control). (J) Quantification for MRE11A protein levels on the cycloheximide chase assay (NS, not significant, **P < 0.01). Error bars represent the mean ± SD from *n* = 3 replicates. Statistical differences were tested using unpaired two‐tailed *t*‐test (B and D) and two‐way ANOVA test and *post hoc* Bonferroni test (H and J).

We first examined the expression of MRE11A and UBQLN4 protein levels in three ESCC cell lines (TE‐4, TE‐8, and TE‐10). Western blot analysis showed that when ESCC cell lines have increased UBQLN4, they consistently have low MRE11A protein levels (Fig. 3A). Ectopic OV of GFP‐tagged UBQLN4 (UBQLN4‐OV) or an EV was performed for TE‐8 and TE‐10 ESCC cell lines. After lentiviral transduction, positive clones were selected, then enriched for GFP‐positive clones using the On‐chip cell sorting system, and finally confirmed for UBQLN4‐OV by western blot (Fig. 3B).

Our hypothesis is that during DNA damage ubiquitinated‐MRE11A levels increased in the DNA damage sites and UBQLN4 may help in promoting the proteasome‐mediated degradation. Thus, we examined the interaction between MRE11A and UBQLN4 using Co‐IP assays. For Co‐IPs, the ESCC cell lines were treated with cisplatin (to enhance the levels of ubiquitinated‐MRE11A and promote the interaction with UBQLN4) and with MG‐132 inhibitor (to block proteasomal degradation). As shown in Fig. 3C,D, UBQLN4 co‐immunoprecipitated with MRE11A in ESCC cell lines. The reciprocal Co‐IP was also performed. MRE11A co‐immunoprecipitated with UBQLN4 in ESCC cell lines (Fig. 3E).

Then, we performed experiments to assess the changes in ubiquitinated‐MRE11A after cisplatin‐induced DNA damage. To do this, we transfected TE‐10 UBQLN4‐OV cells with UBB‐DDK and used anti‐DDK antibody to immunoprecipitate endogenous tagged ubiquitinated‐MRE11A in cisplatin‐treated ESCC cell lines in the presence or absence of MG‐132. To demonstrate that ubiquitinated‐MRE11A was bound to the beads, we pretreated the immunocomplexes with buffer or the recombinant catalytic domain of the de‐ubiquitinase USP2. If the interaction would be mediated by ubiquitination, USP2 would reduce the binding of MRE11A in the immunocomplexes. Ubiquitinated‐MRE11A levels were increased in ESCC cell lines treated with cisplatin in the presence of MG‐132 compared to absence of MG‐132 (Fig. S3A). Moreover, USP2 decreased the amount of ubiquitinated‐MRE11A (Fig. S3A). These results confirmed that cisplatin promotes MRE11A ubiquitination.

Using Co‐IPs, we assessed for ubiquitinated‐MRE11A binding to UBQLN4. To do that, we transfected TE‐10 UBQLN4‐OV cells with UBB‐DDK and used anti‐DDK antibody to co‐immunoprecipitate endogenous ubiquitinated‐MRE11A DDK‐tagged and UBQLN4 in cisplatin‐treated ESCC cell lines in the presence of MG‐132. Ubiquitinated‐MRE11A co‐immunoprecipitated with UBQLN4 in ESCC cell lines (Fig. 3F). These results were further validated using anti‐MRE11A antibody. UBQLN4 co‐immunoprecipitated with ubiquitinated‐MRE11A (Fig. S3B).

In order to analyze MRE11A protein stability in the presence of high UBQLN4 levels, cycloheximide chase assays were performed in UBQLN4‐OV cell lines and compared to respective control EV cell lines. A significant decrease was observed for MRE11A protein levels in UBQLN4‐OV compared to the control EV cell lines (Fig. 3G,H). In IF assays, UBQLN4‐OV cell lines exhibited a reduction in MRE11A expression under basal conditions, but also under cisplatin treatment (Fig. S4A‐F). These observations were further validated using western blot in nuclear fractions obtained from TE‐8 and TE‐10 ESCC cell lines untreated or treated with cisplatin. Endogenous UBQLN4 protein levels increased in the nuclear fractions after cisplatin treatment, while the amount of endogenous MRE11A consistently decreased (Fig. S4G,H). To determine whether MRE11A degradation is mediated by the proteasome, we performed cycloheximide chasing assays in UBQLN4‐OV cell lines in the presence or absence of the proteasome inhibitor MG‐132. We observed that blockage of proteasomal degradation increased the levels of MRE11A (Fig. 3I,J), consistently with the observations as shown Fig. S3A. We concluded that UBQLN4‐OV in ESCC leads to an accelerated MRE11A degradation by targeting ubiquitinated‐MRE11A for proteasomal degradation.

### 3.4 High UBQLN4 leads to worse postoperative survival in ESCCs

**Fig. 4:**
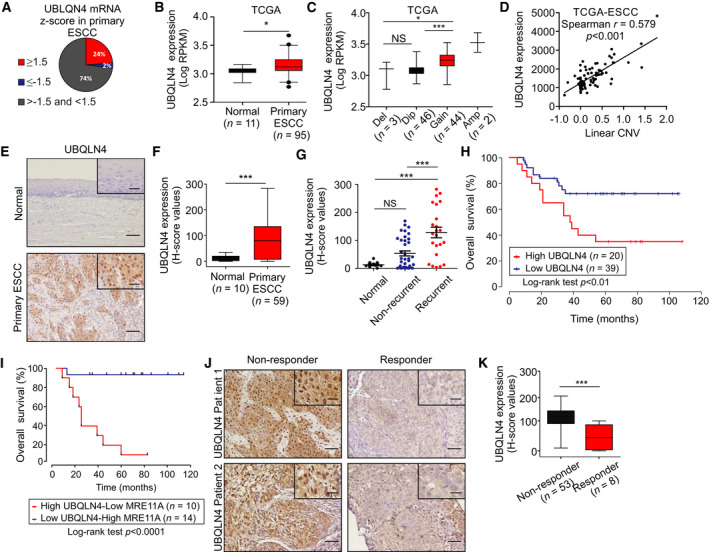
High UBQLN4 leads to worse postoperative survival in ESCC patients. (A) Pie chart showing the proportion ESCC cases with high and low *UBQLN4* mRNA expression levels in the TCGA ESCA database. ESCC patients were divided according to the *z*‐score values into 1) ≥ 1.5, 2) ≤ −1.5, or 3) < 1.5 and > −1.5 for *UBQLN4* mRNA expression levels. (B) Comparison of UBQLN4 mRNA expression levels in normal adjacent esophageal epithelia (*n* = 11) and primary ESCC tumors (*n* = 95) in the TCGA ESCA database (*P < 0.05). (C) The relationship between CNV and *UBQLN4* mRNA expression levels was assessed in primary ESCC tumors using the TCGA ESCA database (*n* = 95) (NS, not significant, *P < 0.05, ***P < 0.001). (D) Correlation between linear CNV and *UBQLN4* mRNA expression levels (Spearman *r* = 0.579, *P* < 0.001). (E) Representative images of UBQLN4 protein levels in normal adjacent esophageal epithelia and primary ESCC tumors stained for UBQLN4 by IHC. Scale bars = 50 µm. Right top insets on each picture show a magnification of MRE11A staining. Scale bars = 10 µm. (F) Comparison of *H*‐scores for UBQLN4 protein levels in normal adjacent esophageal epithelia (*n* = 10) and primary ESCC tumors tissues (*n* = 59) (***P < 0.001). (G) *H*‐scores for UBQLN4 protein levels in normal adjacent esophageal epithelia tissue (*n* = 10), nonrecurrent primary ESCC (*n* = 36), and recurrent primary ESCC tumors (*n* = 23) (NS, not significant, ***P < 0.001). (H) Kaplan–Meier curves comparing OS in ESCC patients with low (*n* = 39) versus high (*n* = 20) UBQLN4 protein levels (*P < *0.01). (I) Kaplan–Meier curves comparing OS in ESCC patients with concurrent low UBQLN4 and high MRE11A (*n* = 14) versus high UBQLN4 and low MRE11A (*n* = 10) protein levels (*P* < 0.001). (J) Representative images of core biopsy tissues from nonresponders (Patients 1 and 2) or responders (Patients 1 and 2) ESCC patients to NAC that were stained for UBQLN4 using IHC. Scale bars = 50 µm. Right top insets on each picture show a magnification of MRE11A staining Scale bars = 10 µm. (K) Comparison of *H*‐scores for UBQLN4 protein levels in core biopsy tissues from nonresponders (*n* = 53) and responders (*n* = 8) ESCC patients to NAC (***P < 0.001). Error bars represent the mean ± SD. Statistical differences were tested using Mann–Whitney test (B), an unpaired two‐tailed *t*‐test with Welch’s correction (F and K), ordinary one‐way ANOVA test and Bonferroni *post hoc* test (C and G), spearman correlation (D), and log‐rank test (H and I).

The Cancer Genome Atlas database analysis revealed that *UBQLN4* was highly expressed (mRNA *z*‐score ≥ 1.5) in about 24% (23 of 95) and decreased in only 2% (mRNA *z*‐score ≤ 1.5) of ESCC patients (Fig. 4A). In addition, ESCC tumors showed significantly higher *UBQLN4* mRNA expression levels compared to normal adjacent esophageal epithelia (Fig. 4B). *UBQLN4* is localized on chromosome 1q23.3, a region commonly amplified in other solid squamous tumor types, and we therefore analyzed the copy number variation (CNV) in this region in primary ESCC tumors using the TCGA ESCA dataset. Forty‐four (48.4%) of the primary ESCC tumors showed a gain in the copy number for the *UBQLN4* gene (Fig. 4C), and also, a significant positive correlation between linear CNV and *UBQLN4* mRNA expression levels was observed (Fig. 4D).

To further elucidate the clinical significance of UBQLN4, IHC and *H*‐score quantification were performed in surgically resected ESCC specimens, in which IHC for MRE11A was also performed (Table 1). Since both nuclear and cytosolic staining for UBQLN4 was positively correlated (Fig. S5A,B), all further analyses were referred to nuclear UBQLN4 staining. UBQLN4 expression in primary ESCC tumors (*n* = 59) was significantly higher than normal adjacent esophageal epithelia (*n* = 10, *H*‐score = 82.0 ± 78.9 versus 12.2 ± 10.2, *P* < 0.0001, Fig. 4E,F). In addition, UBQLN4 expression was significantly higher in recurrent compared to nonrecurrent primary ESCC tumors (Fig. 4G). A cutoff *H*‐score value > 110 was considered as high for UBQLN4 in primary ESCC tumors (Fig. S5A). The survival curve indicated that patients with high UBQLN4 (median 5 years OS = 35 months, *n* = 20) had a significantly worse prognosis compared to those with low UBQLN4 (median 5 years OS = 72 months, *n* = 39, *P* < 0.01; Fig. 4H). Moreover, high UBQLN4 expression determined by IHC staining was an independent factor to predict poor OS in multivariable analysis (*P = *0.02, HR = 3.74, 95% CI, 1.19‐11.76, Table 2). Finally, a comparison of patients with low UBQLN4/high MRE11A (*n* = 14) and high UBQLN4/low MRE11A (*n* = 10) showed that patients with high UBQLN4/low MRE11A have a worse prognosis (*P* < 0.0001, Fig. 4I). These results confirm the role of UBQLN4 in ESCC and reinforce our conclusions on the clinical importance of high UBQLN4/low MRE11A in predicting OS.

IHC analysis for UBQLN4 was also performed for endoscopic biopsy FFPE prior to NAC, which are the same tissues used to assess MRE11A expression. The quantified *H*‐score values demonstrated that UBQLN4 expression was significantly increased in the nonresponder group (*n* = 53) compared to the responder group (*n* = 8) (*H*‐score = 131.8 ± 5.2 versus 60.4 ± 15.7, *P* < 0.001, Fig. 4J,K).

### 3.5 UBQLN4 expression determines the sensitivity to cisplatin in ESCC cell lines

**Fig. 5:**
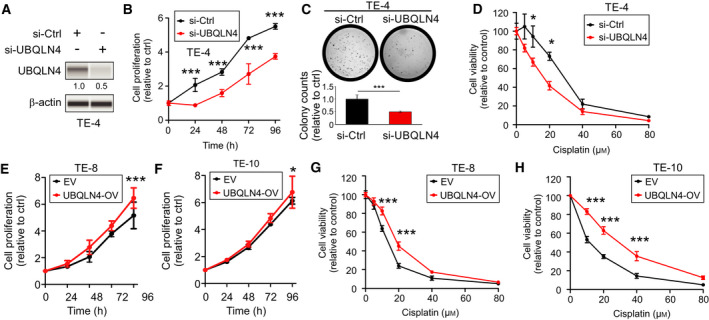
UBQLN4 expression determines the sensitivity to cisplatin in ESCC cell lines. (A) Western blot analysis for UBQLN4 and β‐actin (loading control) in TE‐4 cell lines treated with si‐Ctrl or si‐UBQLN4 (pool siRNA). (B) TE‐4 cell lines were treated with si‐Ctrl or si‐UBQLN4 and cell proliferation was analyzed at indicated time points (***P < 0.001). (C) TE‐4 cell lines were treated with si‐Ctrl or si‐UBQLN4 and analyzed for colony formation. The bar graph showed the quantification of colonies after 12 days of incubation (***P < 0.001). (D) Drug sensitivity assays comparing si‐Ctrl and si‐UBQLN4 in TE‐4 cell lines treated with different cisplatin concentrations (*P < 0.05). (E, F) Cell proliferation assays were performed at indicated time points in TE‐8 (E) and TE‐10 (F) cell lines with EV or UBQLN4‐OV (*P < 0.05, ***P < 0.001). (G, H) Drug sensitivity assays comparing EV and UBQLN4‐OV in TE‐8 (G) and TE‐10 (H) cell lines treated with different cisplatin concentrations (***P < 0.001). Error bars represent the mean ± SD from *n* = 3 replicates. Statistical differences were tested using two‐way ANOVA test and *post hoc* Bonferroni test (B, D, E, F, G, and H) and unpaired two‐tailed *t*‐test (C).

Based on the observation that UBQLN4 expression was increased in patients who did not respond to cisplatin‐based chemotherapy, we hypothesized that UBQLN4 expression may control resistance to cisplatin. Considering a relatively higher UBQLN4 expression, TE‐4 cell lines were selected to perform knockdown experiments. UBQLN4 expression was efficiently reduced in si‐UBQLN4 (pool siRNA) compared to si‐Ctrl‐treated cell lines (Fig. 5A). UBQLN4 knockdown had a suppressive effect in cell proliferation as evaluated by cell viability and colony formation assays (Fig. 5B,C). To test our hypothesis, the effect of UBQLN4 knockdown was examined in drug sensitivity assays by comparing si‐UBQLN4 and si‐Ctrl cell lines. UBQLN4 knockdown cell lines had significantly higher sensitivity to cisplatin (Fig. 5D). Next, UBQLN4‐OV and control EV cell lines were assessed for cell proliferation and cisplatin resistance. UBQLN4‐OV increased cell proliferation (Fig. 5E,F). In drug sensitivity assays, UBQLN4‐OV cell lines presented cisplatin resistance compared to control EV cell lines (Fig. 5G,H). To summarize, UBQLN4 expression level determined the response to cisplatin in ESCC cell lines.

### 3.6 UBQLN4‐OV alleviated DNA damage induced by cisplatin in ESCC cell lines

**Fig. 6:**
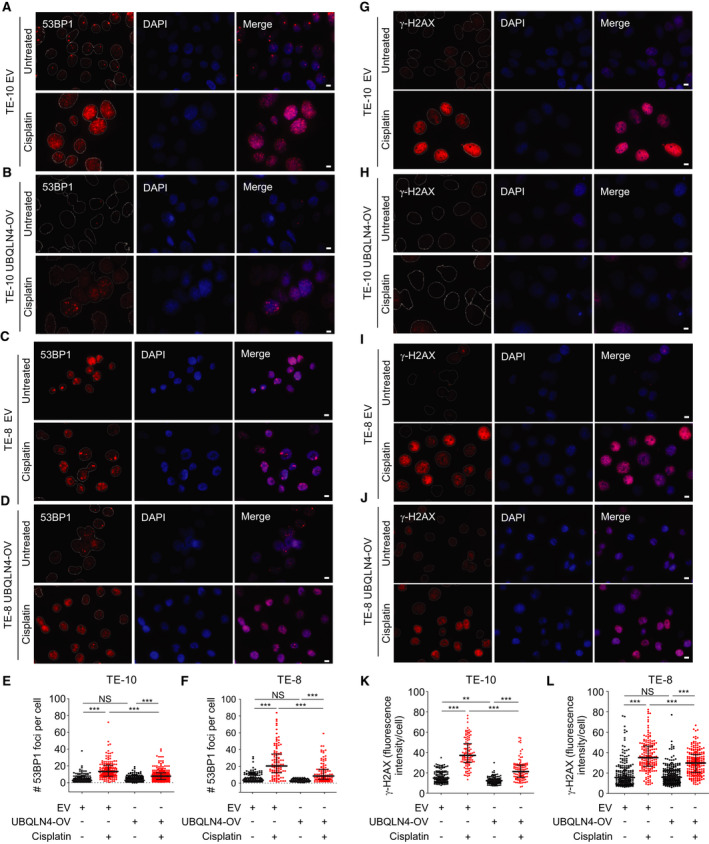
UBQLN4‐OV alleviated DNA damage induced by cisplatin in ESCC cell lines. (A–D) IF staining for 53BP1 was performed in cisplatin‐treated (5 μm, 12 h) or untreated TE‐10 EV (A), TE‐10 UBQLN4‐OV (B), TE‐8 EV (C), and TE‐8 UBQLN4‐OV (D) cell lines. Shown are 53BP1 (red), DAPI (blue), and the merged images. Scale bars: 10 µm. (E, F) Quantification of the number (#) of 53BP1 foci per cell for TE‐10 (E) and TE‐8 (F) cell lines (NS, not significant, ***P < 0.001). (G‐J) IF staining for γ‐H2AX was performed in cisplatin‐treated (5 μm, 12 h) or untreated TE‐10 EV (G), TE‐10 UBQLN4‐OV (H), TE‐8 EV (I), and TE‐8 UBQLN4‐OV (J) cell lines. Shown are γ‐H2AX (red), DAPI (blue), and the merged images. Scale bars = 10 µm. (K‐L) Quantification of γ‐H2AX fluorescence intensity per cell for TE‐10 (K) and TE‐8 (L) cell lines (NS, not significant, **P < 0.01, ***P < 0.001). Error bars represent the mean ± SD from *n* = 3 replicates. Statistical differences were tested using ordinary one‐way ANOVA test and Bonferroni *post hoc* test (E, F, K, and L).

Since UBQLN4 expression determines resistance to cisplatin in ESCC cell lines, we assumed that UBQLN4 may reduce the DNA damage after cisplatin treatment. To determine the DNA damage, two markers (53BP1 and γ‐H2AX) were analyzed by IF in TE‐10 and TE‐8 EV and UBQLN4‐OV cell lines cisplatin‐treated or untreated. As anticipated, ESCC cell lines with UBQLN4‐OV presented a reduction in 53BP1 (Fig. 6A–F) and γ‐H2AX foci formation (Fig. 6G–L) compared to control EV cell lines in both cisplatin‐treated and nontreated conditions. Then, γ‐H2AX was analyzed by IF in si‐Ctrl or si‐UBQLN4 TE‐4 cell lines cisplatin‐treated or untreated. Consistently, TE‐4 cell lines transfected with si‐UBQLN4 showed an enhancement in γ‐H2AX activation (Fig. S6A–C) compared to si‐Ctrl in both cisplatin‐treated and nontreated condition. Finally, we determined whether knockdown of MRE11A also modified the levels of DNA damage induced by cisplatin. Knockdown of MRE11A significantly decreased the levels of γ‐H2AX in cisplatin‐treated conditions (Fig. S6D–F). In order to demonstrate that UBQLN4 localized to DNA damage areas, γ‐H2AX and UBQLN4 were analyzed by IF in si‐UBQLN4 and si‐Ctrl TE‐4 cell lines cisplatin‐treated or untreated. We observed that UBQLN4 localized to DNA damage areas in ESCC cell lines (Fig. S6G) and that γ‐H2AX and UBQLN4 levels positively correlated in TE‐4 cell lines treated with cisplatin (Fig. S6H). In summary, our results reveal that enhanced UBQLN4 expression alleviates the genotoxic stress induced by cisplatin and promotes cisplatin resistance in ESCC cell lines.

## 4 Discussion

This study showed that low MRE11A expression in tumor biopsies taken prior to NAC was associated with cisplatin resistance in ESCC, which was further validated *in vitro* using two cisplatin‐resistant ESCC cell lines. In addition, low MRE11A expression in up‐front surgically resected ESCC tissues was related to significantly worse OS. It is accepted that the loss of MRN complex function results in DNA repair deficiency and increase sensitivity to DNA damaging therapies [17, 18, 22]. However, in certain cancer types, loss of MRE11A and other components of the MRN complex promote a resistant phenotype in response to DNA damage [23, 24, 25]. Recently, new and more complex functions have been assigned to MRE11A in relation to the nuclease activity and the MRN complex [17].

In ESCC patients, UBQLN4 expression was increased and associated with increased gene copy number. Moreover, UBQLN4 protein levels were found to be increased in core biopsies from ESCC patients who have poor response to NAC. Multivariable analysis showed that UBQLN4 was an independent prognostic factor to predict OS. These results suggest a potential role for UBQLN4 to predict cisplatin resistance in ESCC tumors. Additionally, our *in vitro* results demonstrated that UBQLN4 OV alleviated DNA damage induced by cisplatin, while UBQLN4 knockdown caused the opposite effects.

In order to identify molecular mechanisms regulating MRE11A expression at the DSB sites, we interrogated its relationship with UBQLN4. We demonstrated that DNA damage, induced by cisplatin, promotes UBQLN4 increase at DSB in ESCC cell lines. Furthermore, ubiquitinated‐MRE11A interacted with UBQLN4 during DNA damage induced by cisplatin, to be efficiently targeted to the proteasome for degradation. These results validated our previous observations demonstrating that UBQLN4 and ubiquitinated‐MRE11A accumulate at the DSB after inducing DNA damage in other solid tumors [29]. In addition, we previously demonstrated that high UBQLN4 protein levels activate alternative DDR pathways to overcome MRE11A loss and DNA damage induced by cisplatin and other DNA damage drugs; however, further studies are needed to address this and identify those oncogenic pathways reducing DNA damage in ESCC.

UBQLN2 and UBQLN4 have a conserved role as regulators of endoplasmic reticulum stress, autophagy, and mTOR signaling [40]. In regards, UBQLN4 targets nuclear and cytosolic proteins for proteasomal degradation and controls the proteotoxic cell stress [26, 31, 40, 41, 42, 43]. Our studies demonstrated that UBQLN4 levels increased in the nuclear fractions after cisplatin treatment and that also localized at the DSB. Further, we demonstrated that UBQLN4 targets ubiquitinated‐MRE11A for degradation and reduced the genotoxic stress. However, UBLQN4 could potentially regulate the expression of other proteins associated with endoplasmic reticulum stress, autophagy, and mTOR signaling to promote cisplatin resistance in ESCC tumors. These protein interactions and specific pathways activation may also decrease the proteotoxic stress induced by cisplatin. Unraveling the pathways controlled by UBQLN4 in relation to proteotoxic stress may offer new potential targets driving cisplatin resistance and may have synergistic effects with the role of UBQLN4 in controlling DDR.

Various attempts have been made to predict prognosis for patients with locally advanced ESCC after NAC, including diagnostic imaging [44], tumor markers [45], inflammation scores [46], and other clinicopathological factors. In addition, recent molecular studies have shown the role of specific molecules activated during the DDR pathway that confer cisplatin resistance in ESCC [47, 48]. However, the appropriate surrogate marker for treatment response before NAC is still unknown, and none of these markers are currently used in the clinic for early therapeutic decisions. Our study has demonstrated the potential application of MRE11A and UBQLN4 as prognostic markers and potential NAC response markers for patients with ESCC. Future studies will validate the usage of these two biomarkers in prospective NAC clinical trials for ESCC and other solid tumors receiving platinum‐based therapies.

## 5 Conclusion

Decreased MRE11A expression is associated with cisplatin resistance in primary ESCC tumors and cell lines. On contrary, increased copy number for *UBQLN4* gene upregulates UBQLN4 expression in ESCC. Mechanistically, cisplatin treatment increased ubiquitinated‐MRE11A, which is targeted to the proteasome by UBQLN4 and leads to cisplatin resistance *in vitro*. As both MRE11A and UBQLN4 expressions were associated with clinical outcomes, they could serve as predictors for cisplatin‐based NAC response and survival outcomes in patients with primary ESCC tumors.

## Conflict of interest

The authors declare no conflict of interest.

## Author contributions

TM involved in conceptualization and design, methodology, data acquisition, and interpretation (functional and biochemical assays, IHC and *H*‐score), writing—original draft preparation, reviewing, and editing. YS involved in data acquisition, analysis and interpretation (functional and biochemical assays, IF, IHC and *H*‐score), reviewing and editing. TN involved in data acquisition, analysis and interpretation (functional and biochemical assays, IF, IHC, and *H*‐score), reviewing and editing. S‐CC involved in basic statistical and clinical analyses (univariate and multivariable analysis, OS analysis), reviewing, and editing. RDJ involved in data interpretation, reviewing, and editing. SH involved in clinical data analysis, reviewing, and editing. SO involved in clinical data analysis, reviewing, and editing. YS involved in conceptualization and design, reviewing, and editing. HT involved in conceptualization and design, reviewing, and editing. YK involved in conceptualization and design, reviewing, and editing. DSBH involved in conceptualization and design, supervision, funding, writing, reviewing, and editing. MAB involved in conceptualization and design, supervision, methodology, data acquisition, and interpretation (functional and biochemical assays), writing original draft, reviewing, and editing. All authors have read and agreed to publish the current version of the manuscript.

## Supporting information


**Fig. S1**. Staining patterns observed in IHC analysis for MRE11A. A. Representative images are shown for the different staining patterns observed in the ESCC surgical specimens. H‐scores = 0–50; 51–100; 101–150; 151–20; 201–250; 251–300. Scale bars = 50 µm.

**Fig. S2.** MRE11A expression determines cisplatin resistance in ESCC cell lines. A‐B. Western blot analysis for MRE11A, UBQLN4, and β‐actin (loading control) comparing si‐Ctrl and si‐MRE11A (pool siRNA) in TE‐10 (A) and TE‐8 (B) cisplatin‐resistant (Cis‐Res) cell lines. C‐D. Drug sensitivity assays comparing si‐Ctrl or si‐MRE11A (pool siRNA) in TE‐10 (C) and TE‐8 (D) cisplatin‐resistant (Cis‐Res) cell lines treated with different cisplatin concentrations (**P < 0.01, ***P < 0.001). E‐F. Western blot analysis for MRE11A, UBQLN4, and β‐actin (loading control) comparing EV and MRE11A‐OV in TE‐10 (E) or TE‐8 (F) cisplatin‐resistant (Cis‐Res) cell lines. G‐H. Drug sensitivity assays comparing cisplatin‐resistant (Cis‐Res) TE‐10 (G) or TE‐8 (H) cell lines with EV or MRE11A‐OV and treated with different cisplatin concentrations (***P < 0.001). Error bars represent the mean ± SD from *n* = 3 replicates. Statistical differences were tested using two‐way ANOVA test and *post hoc* Bonferroni test (C, D, G, and H).

**Fig. S3.** Ubiquitinated‐MRE11A interacts with UBQLN4. A. IP assay in TE‐10 UBQLN4‐OV and UBB‐OV cell lines that were treated with cisplatin (5 μm) and MG‐132 (5 μm) using anti‐DDK IgG Ab or control IgG Ab. Before elution, the immunocomplexes were treated for 30 min at 37 °C with 50 nm of recombinant human catalytic domain of USP2 or buffer as indicated. MRE11A and ubiquitinated protein levels (ubiquitinated‐MRE11A) were assessed in whole‐cell (WC) lysates and IP fractions (IP). B. Co‐IP assay in TE‐10 UBQLN4‐OV and UBB‐OV cell lines that were treated with cisplatin (5 μm) and MG‐132 (5 μm) using anti‐MRE11A IgG Ab or control IgG Ab. UBQLN4, DDK tag, and MRE11A (loading control) protein levels were assessed in whole‐cell (WC) lysates and Co‐IP fractions (Co‐IP).

**Fig. S4.** UBQLN4 promotes MRE11A degradation in ESCC cell lines. A‐D. Immunofluorescence staining for MRE11A was performed in cisplatin‐treated (5 μm, 12 h) or untreated TE‐10 EV (A), TE‐10 UBQLN4‐OV (B), TE‐8 EV (C), and TE‐8 UBQLN4‐OV (D) cell lines. MRE11A (red), DAPI (blue), and the merged images are shown. Scale bars = 10 µm. E‐F. Quantification of MRE11A fluorescence intensity per cell in TE‐10 (E) and TE‐8 (F) cell lines (*P < 0.05, ***P < 0.001). G‐H. Western blot for UBQLN4, MRE11A, and LSD1 (loading control) in the nuclear fractions isolated from TE‐10 (G) and TE‐8 (H) cell lines that were untreated or treated with cisplatin (5 μm). Error bars represent the mean ± SD from *n* = 3 replicates. Statistical differences were tested using ordinary one‐way ANOVA test and Bonferroni *post hoc* test (E and F).

**Fig. S5**. Staining patterns observed in IHC analysis for UBQLN4, A. Representative images are shown for the different IHC staining patterns observed in the ESCC surgical specimens. H‐scores = 0–50; 51–100; 101–150; 151–20; 201–250; 251–300. Scale bars = 50 µm. B. Correlation of nuclear and cytosolic staining H‐score values for UBQLN4 (Spearman *r* = 0.95, *P* < 0.0001).

**Fig. S6**. Ubiquitinated‐MRE11A interacts with UBQLN4. A‐B. Immunofluorescence staining for γ‐H2AX was performed in cisplatin‐treated (5 μm, 12 h) or untreated TE‐4 si‐Ctrl (A) and si‐UBQLN4 (B) cell lines. γ‐H2AX (green), DAPI (blue), and the merged images are shown. Scale bars = 10 µm. C. Quantification of γ‐H2AX fluorescence intensity per cell in TE‐4 cell lines (*P < 0.05, ***P < 0.001). D‐E. Immunofluorescence staining for γ‐H2AX was performed in cisplatin‐treated (5 μm, 12 h) or untreated TE‐8 si‐Ctrl (D) and si‐MRE11A (E) cell lines. γ‐H2AX (green), DAPI (blue), and the merged images are shown. Scale bars = 10 µm. F. Quantification of γ‐H2AX fluorescence intensity per cell in TE‐8 cell lines (NS not significant, ***P < 0.001). G. Immunofluorescence staining for endogenous UBQLN4 and γ‐H2AX were performed in cisplatin‐treated (5 μm, 12 h) or untreated TE‐4 cell lines. UBQLN4 (red), γ‐H2AX (green), DAPI (blue), and the merged images are shown. Scale bars = 10 µm. H. Correlation between UBQLN4 and γ‐H2AX levels in cisplatin‐treated TE‐4 cell lines (Pearson *r* = 0.74, *P* < 0.001). Error bars represent the mean ± SD from *n* = 3 replicates. Statistical differences were tested using ordinary one‐way ANOVA test and Bonferroni *post hoc* test (C and F).

**Fig. S7**. Western blot uncropped images.

**Fig. S8**. Western blot uncropped images.

**Table S1.** List of antibodies (Ab) and dilutions utilized in this study.

## Data Availability

The Cancer Genome Atlas ESCA datasets available for mRNA expression levels, CNV, and clinical information were obtained in November 2019 at http://cbioportal.org/ and http://firebrowse.org/.

## References

[1] Bray F , Ferlay J , Soerjomataram I , Siegel RL , Torre LA & Jemal A (2018) Global cancer statistics 2018: GLOBOCAN estimates of incidence and mortality worldwide for 36 cancers in 185 countries. CA Cancer J Clin 68, 394–424. 30207593 10.3322/caac.21492

[2] Enzinger PC & Mayer RJ (2003) Esophageal cancer. N Engl J Med 349, 2241–2252. 14657432 10.1056/NEJMra035010

[3] Arnold M , Soerjomataram I , Ferlay J & Forman D (2015) Global incidence of oesophageal cancer by histological subtype in 2012. Gut 64, 381–387. 25320104 10.1136/gutjnl-2014-308124

[4] Smyth EC , Lagergren J , Fitzgerald RC , Lordick F , Shah MA , Lagergren P & Cunningham D (2017) Oesophageal cancer. Nat Rev Dis Primers 3, 17048. 28748917 10.1038/nrdp.2017.48 PMC6168059

[5] Abnet CC , Arnold M & Wei WQ (2018) Epidemiology of esophageal squamous cell carcinoma. Gastroenterology 154, 360–373. 28823862 10.1053/j.gastro.2017.08.023 PMC5836473

[6] van Hagen P , Hulshof MC , van Lanschot JJ , Steyerberg EW , van Berge Henegouwen MI , Wijnhoven BP , Richel DJ , Nieuwenhuijzen GA , Hospers GA , Bonenkamp JJ *et al*. (2012) Preoperative chemoradiotherapy for esophageal or junctional cancer. N Engl J Med 366, 2074–2084. 22646630 10.1056/NEJMoa1112088

[7] Ando N , Kato H , Igaki H , Shinoda M , Ozawa S , Shimizu H , Nakamura T , Yabusaki H , Aoyama N , Kurita A *et al*. (2012) A randomized trial comparing postoperative adjuvant chemotherapy with cisplatin and 5‐fluorouracil versus preoperative chemotherapy for localized advanced squamous cell carcinoma of the thoracic esophagus (JCOG9907). Ann Surg Oncol 19, 68–74. 21879261 10.1245/s10434-011-2049-9

[8] Ter Veer E , Haj Mohammad N , van Valkenhoef G , Ngai LL , Mali RMA , Anderegg MC , van Oijen MGH & van Laarhoven HWM (2016) The efficacy and safety of first‐line chemotherapy in advanced esophagogastric cancer: a network meta‐analysis. J Natl Cancer Inst 108(10), 1–13. 10.1093/jnci/djw166 27576566

[9] Wang T , Yu J , Liu M , Chen Y , Zhu C , Lu L , Wang M , Min L , Liu X , Zhang X *et al*. (2019) The benefit of taxane‐based therapies over fluoropyrimidine plus platinum (FP) in the treatment of esophageal cancer: a meta‐analysis of clinical studies. Drug Des Devel Ther 13, 539–553. 10.2147/DDDT.S189514 PMC6368118 30787595

[10] Ilson DH (2008) Esophageal cancer chemotherapy: recent advances. Gastrointest Cancer Res 2, 85–92. 19259300 PMC2630822

[11] Altorki N & Harrison S (2017) What is the role of neoadjuvant chemotherapy, radiation, and adjuvant treatment in resectable esophageal cancer? Ann Cardiothorac Surg 6, 167–174. 28447006 10.21037/acs.2017.03.16 PMC5387137

[12] Lagergren J , Smyth E , Cunningham D & Lagergren P (2017) Oesophageal cancer. Lancet 390, 2383–2396. 28648400 10.1016/S0140-6736(17)31462-9

[13] Meredith KL , Weber JM , Turaga KK , Siegel EM , McLoughlin J , Hoffe S , Marcovalerio M , Shah N , Kelley S & Karl R (2010) Pathologic response after neoadjuvant therapy is the major determinant of survival in patients with esophageal cancer. Ann Surg Oncol 17, 1159–1167. 20140529 10.1245/s10434-009-0862-1

[14] Samson P , Robinson C , Bradley J , Lockhart AC , Puri V , Broderick S , Kreisel D , Krupnick AS , Patterson GA , Meyers B *et al*. (2016) Neoadjuvant chemotherapy versus chemoradiation prior to esophagectomy: impact on rate of complete pathologic response and survival in esophageal cancer patients. J Thorac Oncol 11, 2227–2237. 27544058 10.1016/j.jtho.2016.07.031 PMC5118087

[15] Tiesi G , Park W , Gunder M , Rubio G , Berger M , Ardalan B , Livingstone A & Franceschi D (2017) Long‐term survival based on pathologic response to neoadjuvant therapy in esophageal cancer. J Surg Res 216, 65–72. 28807215 10.1016/j.jss.2017.03.022

[16] Uemura N & Kondo T (2014) Current status of predictive biomarkers for neoadjuvant therapy in esophageal cancer. World J Gastrointest Pathophysiol 5, 322–334. 25133032 10.4291/wjgp.v5.i3.322 PMC4133529

[17] Syed A & Tainer JA (2018) The MRE11‐RAD50‐NBS1 complex conducts the orchestration of damage signaling and outcomes to stress in DNA replication and repair. Annu Rev Biochem 87, 263–294. 29709199 10.1146/annurev-biochem-062917-012415 PMC6076887

[18] Bian L , Meng Y , Zhang M & Li D (2019) MRE11‐RAD50‐NBS1 complex alterations and DNA damage response: implications for cancer treatment. Mol Cancer 18, 169. 31767017 10.1186/s12943-019-1100-5 PMC6878665

[19] Koczkowska M , Krawczynska N , Stukan M , Kuzniacka A , Brozek I , Sniadecki M , Debniak J , Wydra D , Biernat W , Kozlowski P *et al*. (2018) Spectrum and prevalence of pathogenic variants in ovarian cancer susceptibility genes in a group of 333 patients. Cancers 10, 442. 10.3390/cancers10110442 PMC6266089 30441849

[20] Altan B , Yokobori T , Ide M , Bai T , Yanoma T , Kimura A , Kogure N , Suzuki M , Bao P , Mochiki E *et al*. (2016) High expression of MRE11‐RAD50‐NBS1 is associated with poor prognosis and chemoresistance in gastric cancer. Anticancer Res 36, 5237–5247. 27798884 10.21873/anticanres.11094

[21] Zaki BI , Suriawinata AA , Eastman AR , Garner KM & Bakhoum SF (2014) Chromosomal instability portends superior response of rectal adenocarcinoma to chemoradiation therapy. Cancer 120, 1733–1742. 24604319 10.1002/cncr.28656

[22] Fagan‐Solis KD , Simpson DA , Kumar RJ , Martelotto LG , Mose LE , Rashid NU , Ho AY , Powell SN , Wen YH , Parker JS *et al*. (2020) A P53‐independent DNA damage response suppresses oncogenic proliferation and genome instability. Cell Rep 30, 1385–1399.e7. 32023457 10.1016/j.celrep.2020.01.020 PMC7361372

[23] Choudhury A , Nelson LD , Teo MT , Chilka S , Bhattarai S , Johnston CF , Elliott F , Lowery J , Taylor CF , Churchman M *et al*. (2010) MRE11 expression is predictive of cause‐specific survival following radical radiotherapy for muscle‐invasive bladder cancer. Cancer Res 70, 7017–7026. 20843819 10.1158/0008-5472.CAN-10-1202 PMC2941719

[24] Fan CW , Kopsida M , Liu YB , Zhang H , Gao JF , Arbman G , Cao SY , Li Y , Zhou ZG & Sun XF (2019) Prognostic heterogeneity of MRE11 based on the location of primary colorectal cancer is caused by activation of different immune signals. Front Oncol 9, 1465. 32010608 10.3389/fonc.2019.01465 PMC6979908

[25] Soderlund K , Stal O , Skoog L , Rutqvist LE , Nordenskjold B & Askmalm MS (2007) Intact Mre11/Rad50/Nbs1 complex predicts good response to radiotherapy in early breast cancer. Int J Radiat Oncol Biol Phys 68, 50–58. 17337132 10.1016/j.ijrobp.2006.12.005

[26] Suzuki R & Kawahara H (2016) UBQLN4 recognizes mislocalized transmembrane domain proteins and targets these to proteasomal degradation. EMBO Rep 17, 842–857. 27113755 10.15252/embr.201541402 PMC5278606

[27] Lee DY , Arnott D & Brown EJ (2013) Ubiquilin4 is an adaptor protein that recruits Ubiquilin1 to the autophagy machinery. EMBO Rep 14, 373–381. 23459205 10.1038/embor.2013.22 PMC3615663

[28] Marin I (2014) The ubiquilin gene family: evolutionary patterns and functional insights. BMC Evol Biol 14, 63. 24674348 10.1186/1471-2148-14-63 PMC4230246

[29] Jachimowicz RD , Beleggia F , Isensee J , Velpula BB , Goergens J , Bustos MA , Doll MA , Shenoy A , Checa‐Rodriguez C , Wiederstein JL *et al*. (2019) UBQLN4 represses homologous recombination and is overexpressed in aggressive tumors. Cell 176, 505–519.e22. 30612738 10.1016/j.cell.2018.11.024

[30] Prakash R , Izraely S , Thareja NS , Lee RH , Rappaport M , Kawaguchi R , Sagi‐Assif O , Ben‐Menachem S , Meshel T , Machnicki M *et al*. (2019) Regeneration enhances metastasis: a novel role for neurovascular signaling in promoting melanoma brain metastasis. Front Neurosci 13, 297. 31024232 10.3389/fnins.2019.00297 PMC6465799

[31] Hirayama S , Sugihara M , Morito D , Iemura SI , Natsume T , Murata S & Nagata K (2018) Nuclear export of ubiquitinated proteins via the UBIN‐POST system. Proc Natl Acad Sci USA 115, E4199–E4208. 29666234 10.1073/pnas.1711017115 PMC5939056

[32] Hoshimoto S , Takeuchi H , Ono S , Sim MS , Huynh JL , Huang SK , Marzese DM , Kitagawa Y & Hoon DS (2015) Genome‐wide hypomethylation and specific tumor‐related gene hypermethylation are associated with esophageal squamous cell carcinoma outcome. J Thorac Oncol 10, 509–517. 25514805 10.1097/JTO.0000000000000441

[33] Wang X , Bustos MA , Zhang X , Ramos RI , Tan C , Iida Y , Chang SC , Salomon MP , Tran K , Gentry R *et al*. (2020) Downregulation of the ubiquitin‐E3 ligase RNF123 promotes upregulation of the NF‐κB1 target SerpinE1 in aggressive glioblastoma tumors. Cancers 12, 1081. 10.3390/cancers12051081 PMC7281601 32349217

[34] Bustos MA , Ono S , Marzese DM , Oyama T , Iida Y , Cheung G , Nelson N , Hsu SC , Yu Q & Hoon DSB (2017) MiR‐200a regulates CDK4/6 inhibitor effect by targeting CDK6 in metastatic melanoma. J Invest Dermatol 137, 1955–1964. 28526299 10.1016/j.jid.2017.03.039

[35] Iida Y , Ciechanover A , Marzese DM , Hata K , Bustos M , Ono S , Wang J , Salomon MP , Tran K , Lam S *et al*. (2017) Epigenetic regulation of KPC1 ubiquitin ligase affects the NF‐κB pathway in melanoma. Clin Cancer Res 23, 4831–4842. 28389511 10.1158/1078-0432.CCR-17-0146 PMC5559338

[36] Mackey E , Thelen KM , Bali V , Fardisi M , Trowbridge M , Jordan CL & Moeser AJ (2020) Perinatal androgens organize sex differences in mast cells and attenuate anaphylaxis severity into adulthood. Proc Natl Acad Sci USA 117, 23751–23761. 32917815 10.1073/pnas.1915075117 PMC7519313

[37] Minikel EV , Zhao HT , Le J , O'Moore J , Pitstick R , Graffam S , Carlson GA , Kavanaugh MP , Kriz J , Kim JB *et al*. (2020) Prion protein lowering is a disease‐modifying therapy across prion disease stages, strains and endpoints. Nucleic Acids Res 48, 10615–10631. 32776089 10.1093/nar/gkaa616 PMC7641729

[38] R Core Team (2018) R: A language and environment for statistical computing. R Foundation for Statistical Computing, Vienna, Austria. Available online at https://www.R‐project.org/

[39] Cancer Genome Atlas Research Network; Analysis Working Group: Asan University; BC Cancer Agency; Brigham and Women’s Hospital; Broad Institute; Brown University; Case Western Reserve University; Dana‐Farber Cancer Institute; Duke University; Greater Poland Cancer Centre; Harvard Medical School *et al*. (2017) Integrated genomic characterization of oesophageal carcinoma. Nature 541, 169–175. 28052061 10.1038/nature20805 PMC5651175

[40] Şentürk M , Lin G , Zuo Z , Mao D , Watson E , Mikos AG & Bellen HJ (2019) Ubiquilins regulate autophagic flux through mTOR signalling and lysosomal acidification. Nat Cell Biol 21, 384–396. 30804504 10.1038/s41556-019-0281-x PMC6534127

[41] Edens BM , Yan J , Miller N , Deng HX , Siddique T & Ma YC (2017) A novel ALS‐associated variant in UBQLN4 regulates motor axon morphogenesis. eLife 6, e25453. 28463112 10.7554/eLife.25453 PMC5451210

[42] Davidson JD , Riley B , Burright EN , Duvick LA , Zoghbi HY & Orr HT (2000) Identification and characterization of an ataxin‐1‐interacting protein: A1Up, a ubiquitin‐like nuclear protein. Hum Mol Genet 9, 2305–2312. 11001934 10.1093/oxfordjournals.hmg.a018922

[43] Yu Y , Xu P , Cui G , Xu X , Li K , Chen X & Bao J (2020) UBQLN4 promotes progression of HCC via activating wnt‐β‐catenin pathway and is regulated by miR‐370. Cancer Cell Int 20, 3. 31911755 10.1186/s12935-019-1078-5 PMC6942288

[44] Findlay JM , Bradley KM , Wang LM , Franklin JM , Teoh EJ , Gleeson FV , Maynard ND , Gillies RS & Middleton MR (2017) Metabolic nodal response as a prognostic marker after neoadjuvant therapy for oesophageal cancer. Br J Surg 104, 408–417. 28093719 10.1002/bjs.10435

[45] Okamura A , Matsuda S , Mayanagi S , Kanamori J , Imamura Y , Irino T , Kawakubo H , Mine S , Takeuchi H , Kitagawa Y *et al*. (2020) Clinical significance of pretherapeutic serum squamous cell carcinoma antigen level in patients with neoadjuvant chemotherapy for esophageal squamous cell carcinoma, Ann Surg Oncol 28, 1209–1216. 32524457 10.1245/s10434-020-08716-y

[46] Matsuda S , Takeuchi H , Kawakubo H , Takemura R , Maeda Y , Hirata Y , Kaburagi T , Egawa T , Nishi T , Ogura M *et al*. (2020) Validation study of fibrinogen and albumin score in esophageal cancer patients who underwent esophagectomy: multicenter prospective cohort study. Ann Surg Oncol 28, 774–784. 32737701 10.1245/s10434-020-08958-w

[47] Shi Q , Shen LY , Dong B , Fu H , Kang XZ , Yang YB , Dai L , Yan WP , Xiong HC , Liang Z *et al*. (2018) The identification of the ATR inhibitor VE‐822 as a therapeutic strategy for enhancing cisplatin chemosensitivity in esophageal squamous cell carcinoma. Cancer Lett 432, 56–68. 29890208 10.1016/j.canlet.2018.06.010

[48] Che Y , Wang J , Li Y , Lu Z , Huang J , Sun S , Mao S , Lei Y , Zang R , Sun N *et al*. (2018) Cisplatin‐activated PAI‐1 secretion in the cancer‐associated fibroblasts with paracrine effects promoting esophageal squamous cell carcinoma progression and causing chemoresistance. Cell Death Dis 9, 759. 29988148 10.1038/s41419-018-0808-2 PMC6037765

