# Supplementary figures and images for: Regulation of MRE11A by UBQLN4 leads to cisplatin resistance in patients with esophageal squamous cell carcinoma
Source: Mol Oncol. 2021 Mar 8;15(4):1069–87. doi: 10.1002/1878-0261.12929 (PMC8024730; doi:10.1002/1878-0261.12929)

Figure S1

A

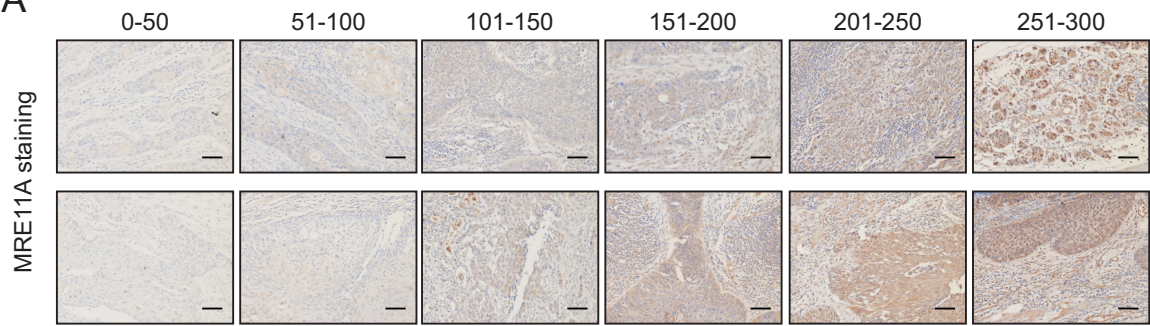

Supplement: Supplementary file 1 — Fig. S1. Staining patterns observed in IHC analysis for MRE11A. A. Representative images are shown for the different staining patterns observed in the ESCC surgical specimens. H‐scores = 0–50; 51–100; 101–150; 151–20; 201–250; 251–300. Scale bars = 50 µm. [file MOL2-15-1069-s003.pdf]

**Figure S2**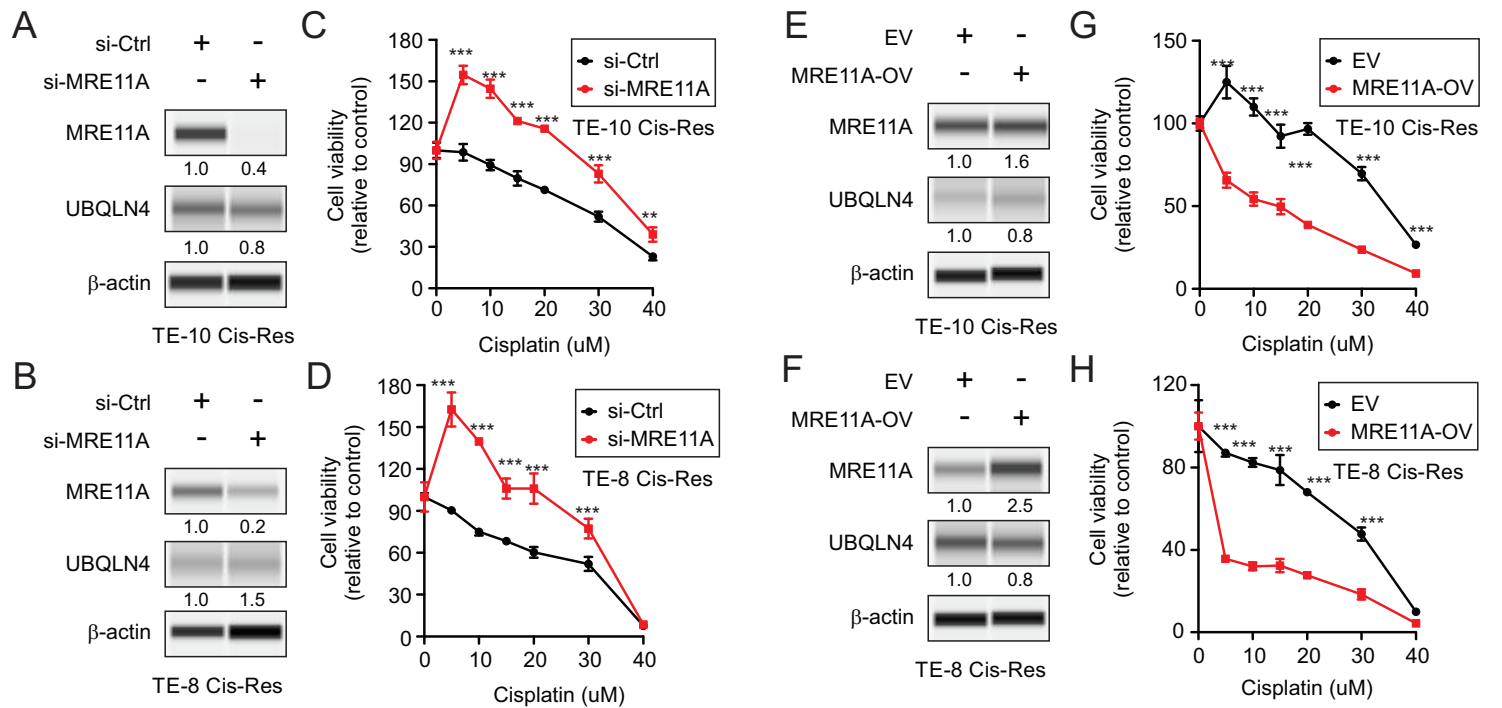

Supplement: Supplementary file 2 — Fig. S2. MRE11A expression determines cisplatin resistance in ESCC cell lines. A‐B. Western blot analysis for MRE11A, UBQLN4, and β‐actin (loading control) comparing si‐Ctrl and si‐MRE11A (pool siRNA) in TE‐10 (A) and TE‐8 (B) cisplatin‐resistant (Cis‐Res) cell lines. C‐D. Drug sensitivity assays comparing si‐Ctrl or si‐MRE11A (pool siRNA) in TE‐10 (C) and TE‐8 (D) cisplatin‐resistant (Cis‐Res) cell lines treated with different cisplatin concentrations (**P < 0.01, ***P < 0.001). E‐F. Western blot analysis for MRE11A, UBQLN4, and β‐actin (loading control) comparing EV and MRE11A‐OV in TE‐10 (E) or TE‐8 (F) cisplatin‐resistant (Cis‐Res) cell lines. G‐H. Drug sensitivity assays comparing cisplatin‐resistant (Cis‐Res) TE‐10 (G) or TE‐8 (H) cell lines with EV or MRE11A‐OV and treated with different cisplatin concentrations (***P < 0.001). Error bars represent the mean ± SD from n = 3 replicates. Statistical differences were tested using two‐way ANOVA test and post hoc Bonferroni test (C, D, G, and H). [file MOL2-15-1069-s005.pdf]

Figure S3

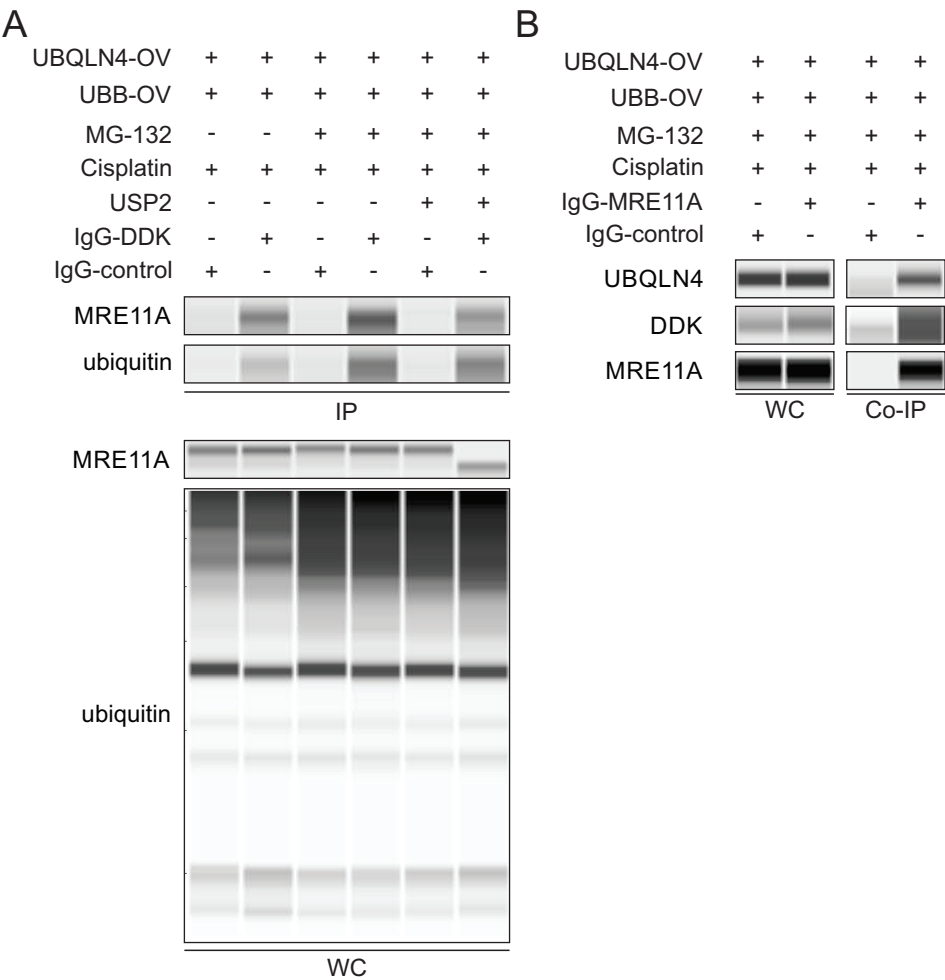

Supplement: Supplementary file 3 — Fig. S3. Ubiquitinated‐MRE11A interacts with UBQLN4. A. IP assay in TE‐10 UBQLN4‐OV and UBB‐OV cell lines that were treated with cisplatin (5 μm) and MG‐132 (5 μm) using anti‐DDK IgG Ab or control IgG Ab. Before elution, the immunocomplexes were treated for 30 min at 37 °C with 50 nm of recombinant human catalytic domain of USP2 or buffer as indicated. MRE11A and ubiquitinated protein levels (ubiquitinated‐MRE11A) were assessed in whole‐cell (WC) lysates and IP fractions (IP). B. Co‐IP assay in TE‐10 UBQLN4‐OV and UBB‐OV cell lines that were treated with cisplatin (5 μm) and MG‐132 (5 μm) using anti‐MRE11A IgG Ab or control IgG Ab. UBQLN4, DDK tag, and MRE11A (loading control) protein levels were assessed in whole‐cell (WC) lysates and Co‐IP fractions (Co‐IP). [file MOL2-15-1069-s004.pdf]

Figure S4

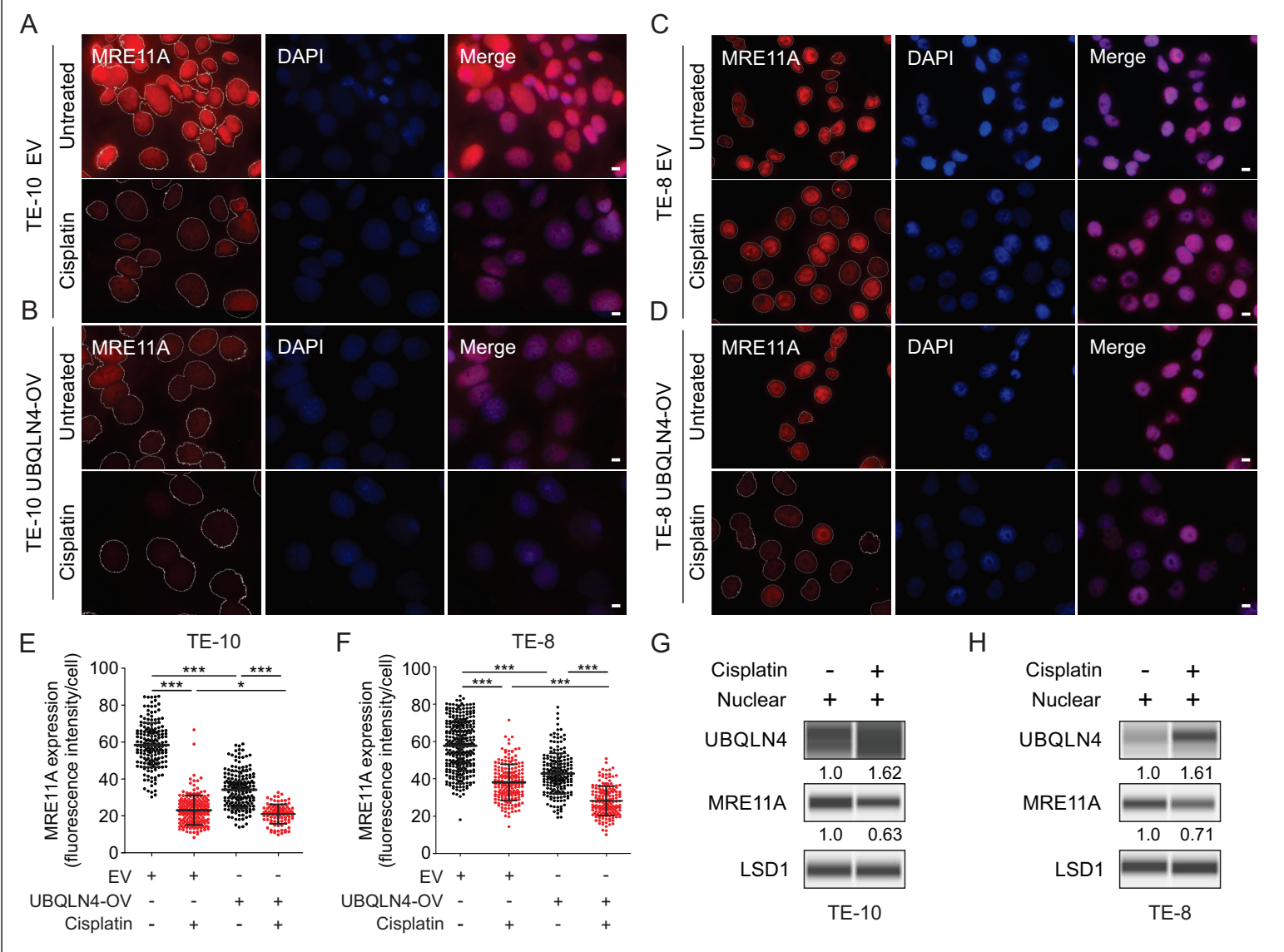

Supplement: Supplementary file 4 — Fig. S4. UBQLN4 promotes MRE11A degradation in ESCC cell lines. A‐D. Immunofluorescence staining for MRE11A was performed in cisplatin‐treated (5 μm, 12 h) or untreated TE‐10 EV (A), TE‐10 UBQLN4‐OV (B), TE‐8 EV (C), and TE‐8 UBQLN4‐OV (D) cell lines. MRE11A (red), DAPI (blue), and the merged images are shown. Scale bars = 10 µm. E‐F. Quantification of MRE11A fluorescence intensity per cell in TE‐10 (E) and TE‐8 (F) cell lines (*P < 0.05, ***P < 0.001). G‐H. Western blot for UBQLN4, MRE11A, and LSD1 (loading control) in the nuclear fractions isolated from TE‐10 (G) and TE‐8 (H) cell lines that were untreated or treated with cisplatin (5 μm). Error bars represent the mean ± SD from n = 3 replicates. Statistical differences were tested using ordinary one‐way ANOVA test and Bonferroni post hoc test (E and F). [file MOL2-15-1069-s007.pdf]

**Figure S5**

**A**

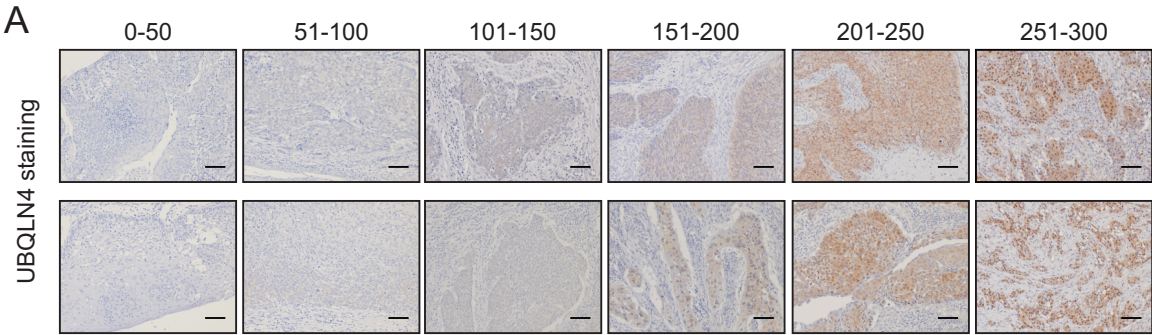

**B**

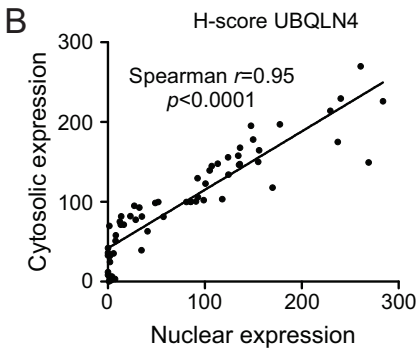

Supplement: Supplementary file 5 — Fig. S5. Staining patterns observed in IHC analysis for UBQLN4, A. Representative images are shown for the different IHC staining patterns observed in the ESCC surgical specimens. H‐scores = 0–50; 51–100; 101–150; 151–20; 201–250; 251–300. Scale bars = 50 µm. B. Correlation of nuclear and cytosolic staining H‐score values for UBQLN4 (Spearman r = 0.95, P < 0.0001). [file MOL2-15-1069-s008.pdf]

Figure S6

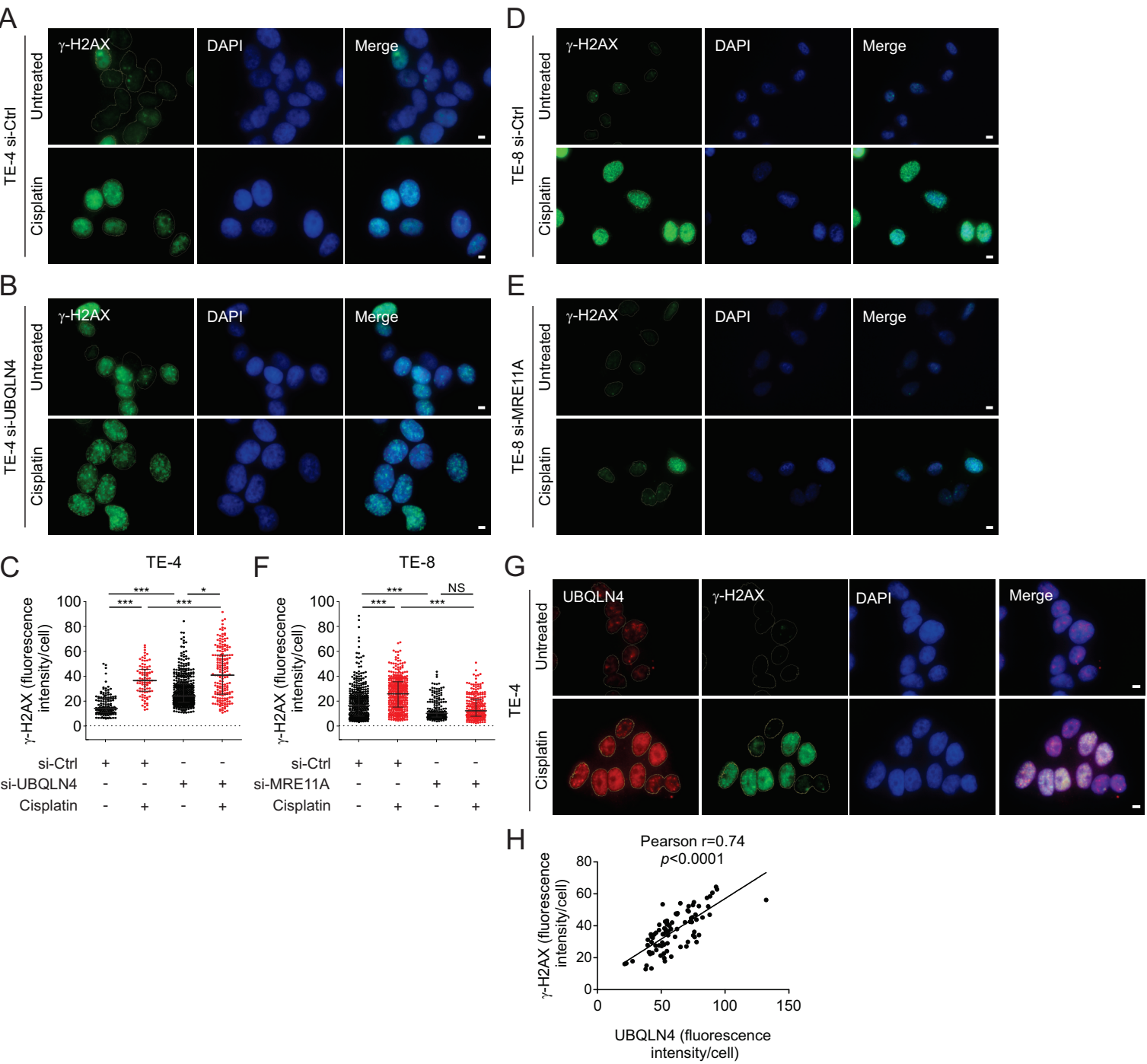

Supplement: Supplementary file 6 — Fig. S6. Ubiquitinated‐MRE11A interacts with UBQLN4. A‐B. Immunofluorescence staining for γ‐H2AX was performed in cisplatin‐treated (5 μm, 12 h) or untreated TE‐4 si‐Ctrl (A) and si‐UBQLN4 (B) cell lines. γ‐H2AX (green), DAPI (blue), and the merged images are shown. Scale bars = 10 µm. C. Quantification of γ‐H2AX fluorescence intensity per cell in TE‐4 cell lines (*P < 0.05, ***P < 0.001). D‐E. Immunofluorescence staining for γ‐H2AX was performed in cisplatin‐treated (5 μm, 12 h) or untreated TE‐8 si‐Ctrl (D) and si‐MRE11A (E) cell lines. γ‐H2AX (green), DAPI (blue), and the merged images are shown. Scale bars = 10 µm. F. Quantification of γ‐H2AX fluorescence intensity per cell in TE‐8 cell lines (NS not significant, ***P < 0.001). G. Immunofluorescence staining for endogenous UBQLN4 and γ‐H2AX were performed in cisplatin‐treated (5 μm, 12 h) or untreated TE‐4 cell lines. UBQLN4 (red), γ‐H2AX (green), DAPI (blue), and the merged images are shown. Scale bars = 10 µm. H. Correlation between UBQLN4 and γ‐H2AX levels in cisplatin‐treated TE‐4 cell lines (Pearson r = 0.74, P < 0.001). Error bars represent the mean ± SD from n = 3 replicates. Statistical differences were tested using ordinary one‐way ANOVA test and Bonferroni post hoc test (C and F). [file MOL2-15-1069-s006.pdf]

Figure S7

Fig.2A, E

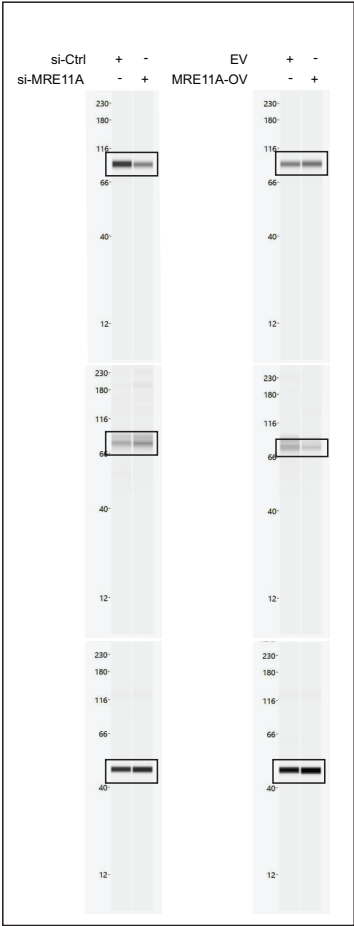

Fig. 2K-M

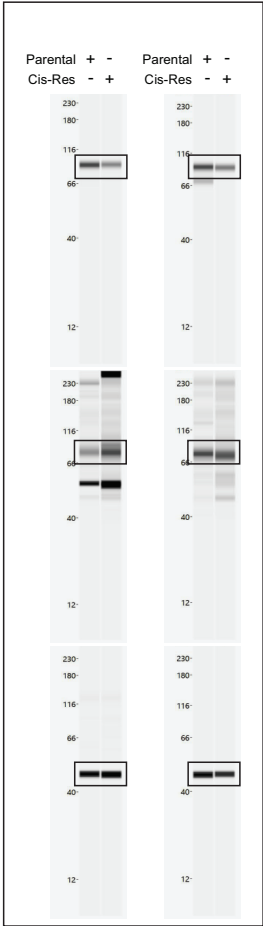

Fig. 3B

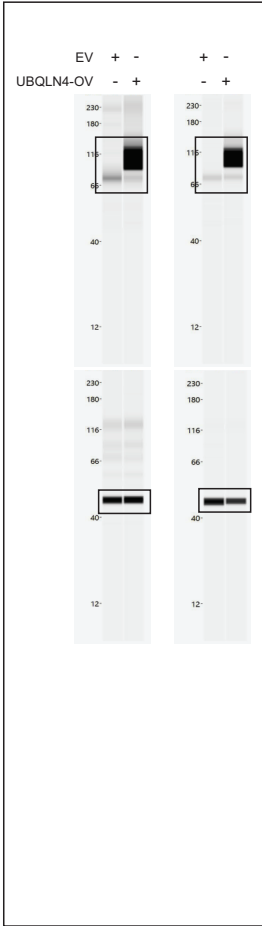

Fig.3E

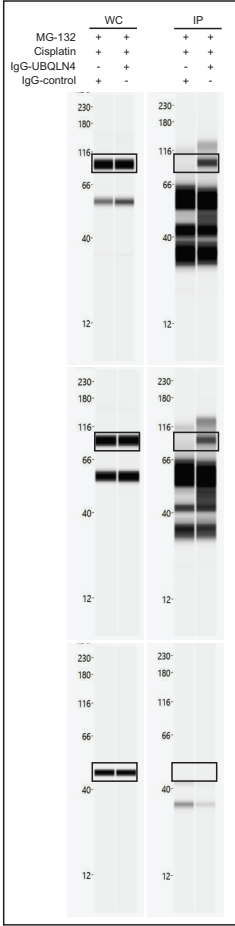

Fig. 3G

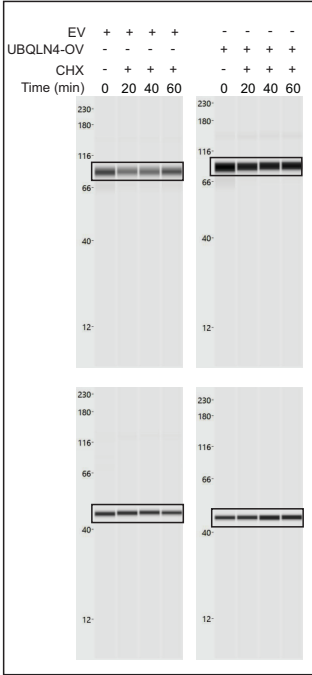

Fig. 2B, F

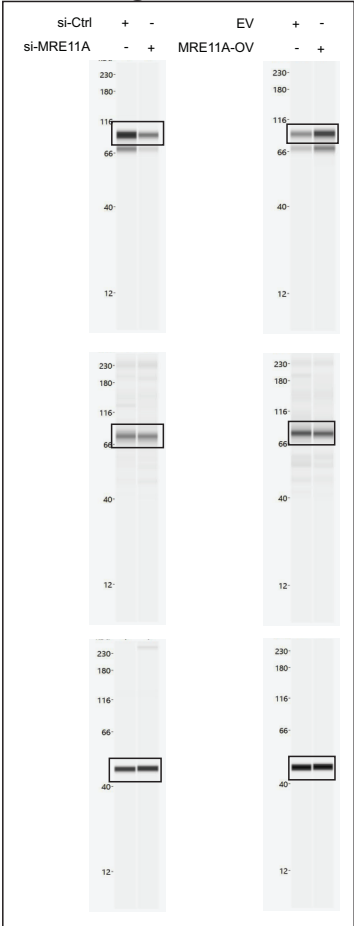

Fig.3A

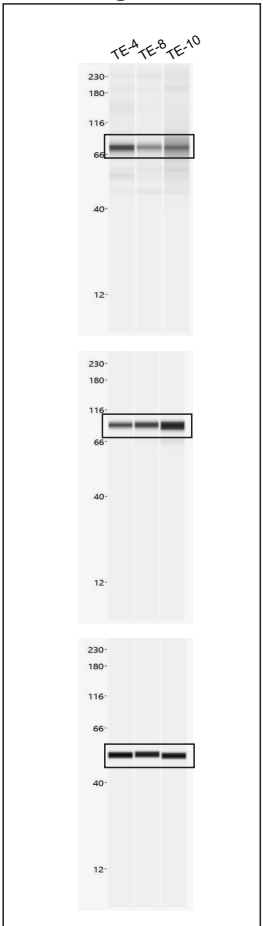

Fig. 3C

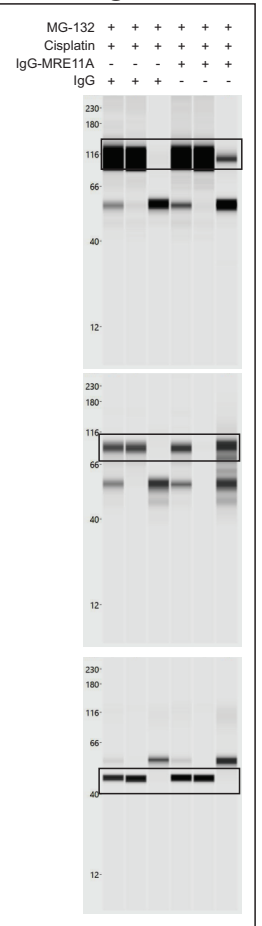

Fig.3F

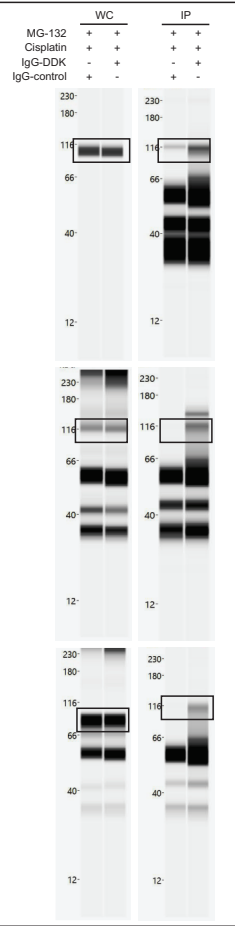

Fig. 3I

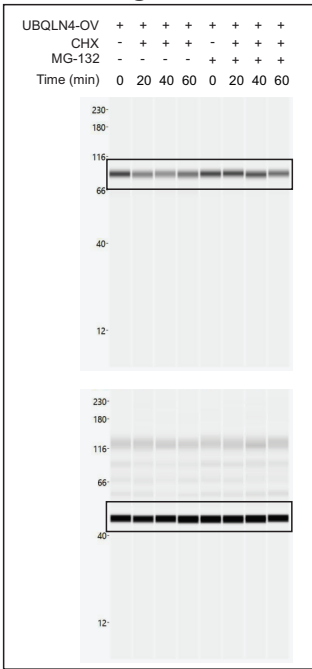

Supplement: Supplementary file 7 — Fig. S7. Western blot uncropped images. [file MOL2-15-1069-s002.pdf]

Figure S8

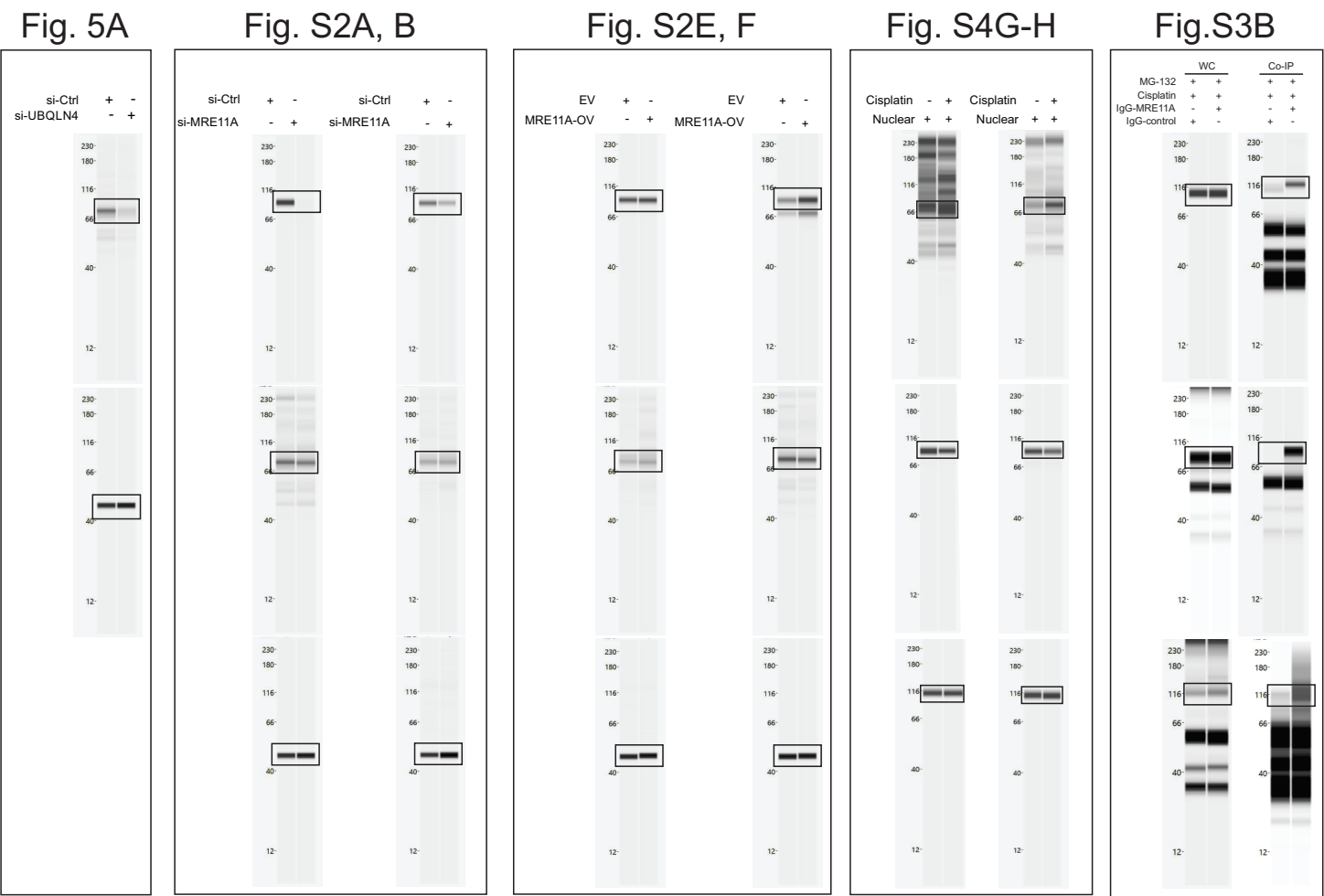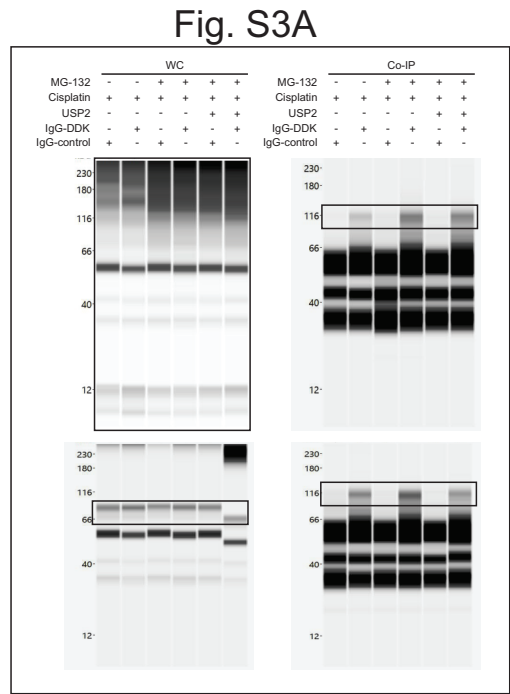

Supplement: Supplementary file 8 — Fig. S8. Western blot uncropped images. [file MOL2-15-1069-s001.pdf]
